# GAMMI: graph-guided contrastive and adversarial integration of single-cell and spatial multi-omics data

**DOI:** 10.1093/bib/bbag218

**Published:** 2026-05-11

**Authors:** Yipei Yu, Meihua Long, Jiali Song, Kai Cao, Meimei Luo, Wei Liu, Zhiwei Rong, Yan Hou

**Affiliations:** Department of Biostatistics, School of Public Health, Peking University Health Science Center, No. 38 Xueyuan Road, Haidian District, Beijing 100191, China; Department of Biostatistics, School of Public Health, Peking University Health Science Center, No. 38 Xueyuan Road, Haidian District, Beijing 100191, China; Department of Biostatistics, School of Public Health, Peking University Health Science Center, No. 38 Xueyuan Road, Haidian District, Beijing 100191, China; Department of Biostatistics, School of Public Health, Peking University Health Science Center, No. 38 Xueyuan Road, Haidian District, Beijing 100191, China; Department of Biostatistics, School of Public Health, Peking University Health Science Center, No. 38 Xueyuan Road, Haidian District, Beijing 100191, China; Department of Biostatistics, School of Public Health, Peking University Health Science Center, No. 38 Xueyuan Road, Haidian District, Beijing 100191, China; Department of Biostatistics, School of Public Health, Peking University Health Science Center, No. 38 Xueyuan Road, Haidian District, Beijing 100191, China; School of Public Health, Tianjin Medical University, No. 22 Qixiangtai Road, Heping District, Tianjin 300203, China; Department of Biostatistics, School of Public Health, Peking University Health Science Center, No. 38 Xueyuan Road, Haidian District, Beijing 100191, China; Peking University Cancer Hospital and Institute, No. 52 Fucheng Road, Haidian District, Beijing 100142, China; Peking University Clinical Research Center, Peking University, No. 38 Xueyuan Road, Haidian District, Beijing 100191, China

**Keywords:** single-cell multi-omics integration, spatial transcriptomics, graph neural network, mosaic integration

## Abstract

Integrating single-cell and spatial multi-omics data is essential for resolving cellular identity, regulatory programs and tissue organization, yet remains challenging under realistic experimental designs. In practice, data are often incomplete, unpaired and partially overlapping across batches and platforms, resulting in mosaic settings where missingness, batch effects and limited correspondence are tightly coupled. Existing methods typically rely on shared cells, explicit anchors or post hoc mapping, and can become unstable as overlap diminishes or spatial structure is not explicitly modeled. Here we present GAMMI (Graph-guided Adversarial Mosaic Multi-omics Integration), a unified graph learning framework for mosaic integration of heterogeneous single-cell and spatial multi-omics data. Rather than performing direct cell or sample alignment, GAMMI learns biologically meaningful relational structure by jointly embedding cells and molecular features in a shared latent space using heterogeneous graphs that encode cell–feature, feature–feature and spatial adjacency relationships. An edge-based contrastive objective with missingness-aware negative sampling avoids false-negative supervision under unobserved modalities, while adversarial domain adaptation suppresses batch-associated variation at the embedding level. Spatial data are incorporated as structurally informative constraints during learning, enabling systematic enrichment of molecular representations across spatial locations. Across diverse mosaic single-cell benchmarks and spatial datasets, GAMMI consistently outperforms state-of-the-art methods in biological conservation, batch correction and spatial reconstruction, including in low-overlap and fully unpaired regimes.

## Introduction

Advances in single-cell omics technologies have enabled high-resolution profiling of molecular states at the level of individual cells, providing systematic insights into cellular heterogeneity and regulatory mechanisms [1–3]. However, single-cell data alone do not capture the spatial organization of cells within tissues or their local microenvironment. In contrast, spatial omics technologies preserve tissue architecture and provide essential information on cellular positions and neighborhood structure [4]. Relying solely on single-cell data limits the study of spatially organized biological processes, while spatial data alone are often constrained by limited molecular coverage and statistical power. Consequently, jointly modeling single-cell molecular profiles together with spatial structure has become a critical strategy for understanding tissue function and disease mechanisms [2, 5].

In practical applications, spatial omics experiments are constrained by throughput, technical complexity, and cost, and therefore typically yield a limited number of spatial locations with restricted molecular measurements. Such datasets alone are often insufficient for robust inference of cell states or regulatory programs. A natural and necessary strategy is to incorporate high-throughput single-cell data to enhance the molecular resolution and statistical power of spatial analyses. Moreover, comprehensive characterization of cellular states frequently requires multiple molecular modalities, such as chromatin accessibility or protein abundance. However, in real-world settings, it is rarely possible to obtain large-scale single-cell multi-omics datasets that are strictly paired with the target spatial samples. Instead, the most accessible data are often small, heterogeneous datasets collected across different batches, experimental conditions, or studies. Aggregating these partially overlapping datasets leads to mosaic multi-omics configurations, which represent a realistic and practical source of complementary molecular information [5]. Methods designed for real applications must therefore be able to robustly exploit mosaic multi-omics data under limited overlap and strong heterogeneity to augment spatial analyses. Together, these considerations define a central challenge: how to systematically augment spatial analyses using heterogeneous, unpaired and mosaic single-cell multi-omics data without relying on strong correspondence or complete observations.

Most existing single-cell–spatial integration methods focus on a single modality, typically transcriptomics, and aim to map single-cell expression profiles onto spatial coordinates to reconstruct tissue organization or infer cellular composition. Representative strategies include label transfer or deconvolution that assigns single-cell-defined populations to spatial locations based on expression similarity [6], as well as distribution alignment approaches that minimize discrepancies between single-cell and spatial expression profiles to generate spatial mappings [7, 8]. While successful in transcriptome-only settings, these methods typically assume that spatial measurements contain sufficient molecular information for reliable mapping and that shared expression structure or anchors exist between single-cell and spatial data—assumptions often violated in practice because spatial datasets are small, molecularly sparse, and rarely provide explicit one-to-one correspondences. In contrast, single-cell multi-omics integration has become a mature and active area, aiming to learn consistent low-dimensional representations across modalities through latent-variable and matrix factorization models [9], manifold alignment and optimal transport approaches [10], deep generative and contrastive frameworks [11–13], and more recently graph-based methods that explicitly model relationships between cells or molecular features [14–16]. However, these two lines of work address complementary but separate aspects of the problem: single-cell–spatial methods are mainly designed for transcriptome-centered spatial mapping, whereas single-cell multi-omics methods are mainly optimized for cross-modality alignment without explicitly incorporating spatial structure as a constraint during representation learning. As a result, a key gap remains under realistic settings where spatial information, mosaic missingness, unpaired observations, and batch heterogeneity co-occur. A summary comparison of representative single-cell and spatial multi-omics integration methods, including their support for unpaired, mosaic, missing-modality, graph-based, and spatial settings, is provided in Supplementary Table S6.

To address these challenges, we introduce GAMMI (Graph-Guided Contrastive and Adversarial Integration of Single-cell and Spatial Multi-omics Data), a unified framework that reformulates single-cell and spatial multi-omics integration as a problem of heterogeneous relational representation learning under realistic mosaic settings. Rather than focusing on cell or sample alignment, GAMMI explicitly learns biologically meaningful relational structures—including cell–feature, feature–feature, and spatial neighborhood relationships—through heterogeneous graph modeling. To prevent spurious supervision induced by missing data, GAMMI distinguishes unmeasured entries from true zeros and introduces negative examples only when observations are confirmed, thereby improving stability under severe missingness. GAMMI further treats spatial omics as a structurally informative but molecularly limited modality and incorporates it as a structural constraint during representation learning, rather than using spatial information only as a post hoc mapping target. By using molecular features as mediators for cross-modal information propagation, GAMMI enables robust alignment and biological discovery even in the absence of shared cells or shared modalities. Across multiple single-cell mosaic benchmarks, spatial multi-omics datasets, and disease-related data, GAMMI consistently achieves more robust integration, preserving biological structure while improving cross-batch and cross-modality consistency, particularly under low-overlap and high-missingness conditions. Moreover, the unified latent space supports direct discovery of cross-modal regulatory associations and spatial markers, highlighting the practical value of GAMMI for analyzing complex tissues under realistic experimental constraints.

## Result

### The GAMMI workflow

**Figure 1:**
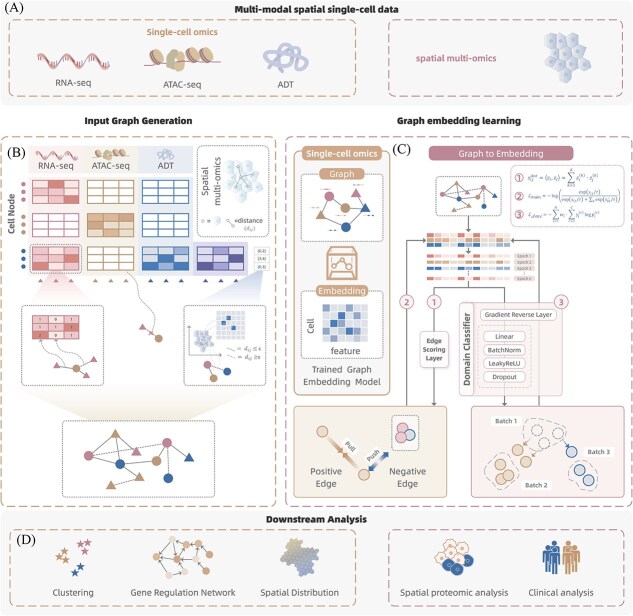
Overview of the GAMMI framework. (a) GAMMI takes multi-modal single-cell and spatial omics data as input, including mosaic single-cell modalities (scRNA-seq, scATAC-seq, and ADT) and spatial multi-omics data. (b) A unified heterogeneous graph is constructed, where nodes represent cells and features, and edges encode cell–feature associations, spatial cell–cell proximity, and optional feature–feature prior relationships. (c) Joint embeddings are learned via edge-based contrastive learning combined with adversarial domain adaptation to obtain batch-robust representations. (d) The learned embeddings support downstream analyses such as clustering, gene regulatory network inference, spatial pattern reconstruction, and clinical analysis.

To address the challenges of integrating mosaic and spatial single-cell multi-omics data with missing modalities and batch heterogeneity, we propose GAMMI, a unified graph-based embedding framework. GAMMI constructs a heterogeneous graph that jointly models cells and molecular features across multiple omics layers, incorporating biologically informed edge types including expression-derived, spatial proximity-based, and feature-feature associations (Fig. 1a and b). This graph is then embedded into a shared low-dimensional space using a contrastive learning strategy that separates true and perturbed edges, and an adversarial domain alignment module that mitigates batch effects (Fig. 1c). The resulting representations support a range of downstream tasks such as cell clustering, spatial pattern discovery, gene regulatory network inference, and clinical analysis.

### Comprehensive evaluation of multi-omics integration methods on five mosaic single-cell datasets

**Figure 2:**
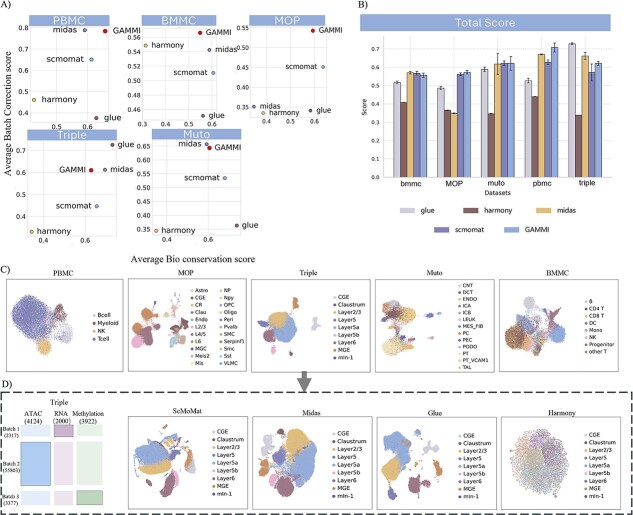
Comprehensive benchmarking of GAMMI and baseline methods across five mosaic multi-omics datasets. (a) Scatter plots comparing biological conservation (x-axis) and batch correction (y-axis) for each dataset. GAMMI consistently occupies the upper-right quadrant, indicating balanced performance across both integration dimensions. (b) Bar plots of total integration scores across all methods and datasets. (c) UMAPs of GAMMI embeddings colored by cell types, showing effective integration and biological separation. (d) UMAPs of the Triple dataset using baseline methods. GAMMI outperforms others in preserving structure across unshared modalities.

To systematically assess the robustness and generalizability of GAMMI, we curated a benchmark composed of five representative mosaic single-cell multi-omics datasets: peripheral blood mononuclear cells (PBMC), mouse brain cortex (MOP), bone marrow mononuclear cells (BMMC), Muto, and adult mouse cortical neuron cell dataset (Triple) (Fig. 2). These datasets span a broad spectrum of integration complexity, ranging from fully shared modalities to completely unpaired omics layers. PBMC contains a shared protein modality across all batches, providing a strong anchor for integrating RNA and assay for transposase-accessible chromatin (ATAC) modalities. In contrast, MOP includes only one batch with full modality coverage, simulating minimal-anchor scenarios. BMMC features an antibody-derived tag (ADT) modality shared across batches, but with very few features, representing a low-information anchor challenge. Muto and Triple exemplify the most difficult cases, where no modality is shared across batches. In Muto, each batch contains a distinct RNA–ATAC pair. In Triple, data are drawn from three completely independent studies measuring RNA, ATAC, and DNA methylation separately, forming a highly heterogeneous and realistic diagonal integration setting.

We benchmarked GAMMI against four state-of-the-art methods, GLUE [14], Harmony [17], MIDAS [18], and scMoMaT [19], using eleven widely adopted metrics covering biological structure preservation and batch effect removal. As shown in Fig. 2a, GAMMI consistently achieved superior performance across all datasets, with lower variance and higher average scores in both categories. In particular, GAMMI demonstrated notable robustness in the most challenging cases (Muto and Triple), where other methods exhibited a marked drop in total performance. Notably, performance degradation in baseline methods coincides with scenarios where shared modalities or anchor batches are absent, highlighting their reliance on direct alignment or anchor-supported propagation. By contrast, GAMMI maintains stable performance by enabling cross-modality information flow through feature-mediated graph structure, independent of shared cells or modalities. An ablation analysis of key GAMMI components and hyperparameters further supports the contribution of the proposed design choices to overall integration performance (Supplementary Table S1). GAMMI also maintained strong scores on datasets with minimal anchors (MOP) and sparse-feature anchors (BMMC), outperforming methods that rely heavily on shared modality overlap. Detailed metric values and method-wise averages are available in Supplementary Figs S1–S7. In addition, GAMMI achieves consistently strong batch-effect removal across all five datasets, and the corresponding visualization results are provided in Supplementary Figs S14–S18.

To visually inspect integration outcomes, Fig. 2b shows UMAP embeddings generated by GAMMI across the five datasets. The results reveal effective alignment of batches and preservation of biologically meaningful cell type separation across diverse configurations. In the most challenging Triple dataset, GAMMI successfully integrated cells across three distinct omics layers, producing coherent and continuous manifolds that preserve cross-modality biological structure. In comparison, Fig. 2c illustrates the UMAP results of the Triple dataset obtained using baseline methods. Most baseline methods exhibit modality-driven fragmentation, where cells cluster primarily by their omics origin rather than biological identity. Specifically, scMoMaT [19] and Harmony [17] tend to separate cells by modality, causing the same cell types to fragment across layers. MIDAS [18] and GLUE [14] further fail to propagate cross-modality information under fully unpaired conditions, resulting in discontinuous manifolds and broken trajectories that hinder biological interpretation. For completeness, we also evaluated MultiVI, a variational framework that is not fully compatible with all mosaic configurations. Its UMAP visualizations are shown in Supplementary Figs S11, S12, and S13, and the corresponding quantitative metrics for the MOP and Muto datasets are reported in Supplementary Tables S2 and S3.

Together, these results demonstrate that GAMMI provides consistently strong integration performance across a wide range of mosaic configurations. Its use of heterogeneous graph construction, bilinear embedding transformation, and domain-adversarial training with randomized batch vector augmentation enables it to effectively capture biological structure while removing technical variation, even in the absence of shared modalities or anchor batches.

### GAMMI illustrates AD-related neural development and the underlying regulatory mechanism

To further evaluate the utility of our GAMMI framework, we applied it to the scRNA-seq dataset GSE138852, comprising 13 214 single nuclei collected from six Alzheimer’s disease (AD) patients and six matched healthy controls.

**Figure 3:**
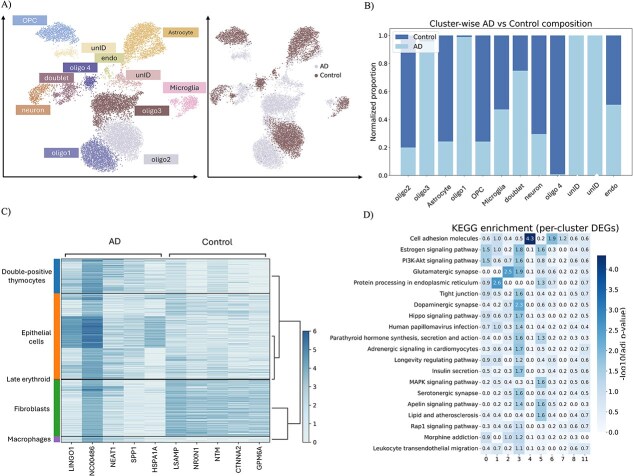
Alzheimer’s disease data set (GSE138852) analysis based on GAMMI. (a) UMAP embedding of cells colored by annotated cell types (left) and AD/control condition (right). (b) Cell-type composition of AD and control samples, computed on a per-individual basis, showing increased proportions of astrocytes and the oligo3 subpopulation in AD. (c) Heatmap of top DEGs across major cell types confirms clear expression signatures. (d) KEGG pathway enrichment across GAMMI clusters reveals functional heterogeneity.

Using GAMMI embeddings followed by Leiden clustering, we identified 12 distinct cell populations, including microglia, astrocytes, neurons, oligodendrocyte progenitor cells (OPCs), and multiple oligodendrocyte subclusters (Fig. 3a). UMAP visualization showed that these cell types were clearly separated in the embedding space, with notable shifts in the spatial distribution between AD and control samples. Specifically, we observed an increased trend in the relative abundance of astrocytes and the oligodendrocyte subcluster oligo3 in AD patients, whereas OPCs and microglia were slightly more represented in controls (Fig. 3b), suggesting potential AD-associated cellular composition changes. Statistical comparisons of cell-type and subcluster proportions between AD and control samples are summarized in Supplementary Table S5. Importantly, these disease-associated patterns emerge from a unified embedding learned without relying on predefined anchors or cell-type labels, highlighting GAMMI’s ability to preserve biologically meaningful structure under heterogeneous and batch-confounded conditions.

To further characterize disease-associated transcriptional changes, we performed differential expression analysis between AD and control samples within each major cell type. The top differentially expressed genes (DEGs) displayed clear, cell-type-specific disease signatures (Fig. 3c), confirming the biological relevance of the GAMMI-derived clusters. In addition, established AD risk genes, including APP, CLU, BACE1, PSEN1, MAPT, and SORL1, showed cluster-specific expression patterns across GAMMI-derived clusters (Supplementary Fig. S8). Pathway enrichment analysis based on the top 100 DEGs per cluster further revealed functional heterogeneity across clusters, including enrichment in synaptic signaling (e.g. glutamatergic synapse, long-term potentiation), endoplasmic reticulum stress, oxidative phosphorylation, and calcium signaling pathways (Fig. 3d). Additional KEGG enrichment analysis of AD-associated differentially expressed genes further highlighted pathways related to endoplasmic reticulum stress, lysosomal function, steroid biosynthesis, antigen processing, cell adhesion, and phosphatidylinositol signaling (Supplementary Fig. S9). Notably, several of these pathways are strongly implicated in neurodegenerative processes, highlighting the potential of GAMMI to uncover both cell-type-specific structure and underlying molecular programs in AD.

### Practical feasibility verification in real-world scenarios

To further assess the practical feasibility of GAMMI in real-world settings, we constructed a composite integration scenario using heterogeneous single-cell and spatial transcriptomics datasets from mouse cortex. Specifically, the single-cell RNA-seq and scATAC-seq data were obtained from a published study, whereas the spatial transcriptomics data were collected from two independent mouse cortex batches. This setting more closely reflects realistic biological studies, in which datasets are often generated from different experiments, platforms, or cohorts and therefore exhibit incomplete modality overlap, batch effects, and biological heterogeneity, thereby providing a more realistic test bed beyond idealized benchmark settings.

Under this heterogeneous and imperfect configuration, GAMMI was still able to jointly integrate transcriptomic, chromatin accessibility, and spatial information within a unified framework. Rather than relying on strict one-to-one correspondence across datasets or complete modality availability, GAMMI learned a shared representation that aligned information across modalities and spatial batches while preserving biologically meaningful structure. These results show that the framework remains applicable even when the input data deviate from idealized benchmark assumptions.

This scenario also involves several challenges commonly encountered in practice, including cross-dataset batch variation, differences in observation granularity between single-cell and spatial measurements, partial modality missingness, and biological variability across spatial batches. GAMMI addresses these challenges by jointly modeling cells and molecular features in a unified heterogeneous graph and enabling cross-modality information propagation through relational structure learning. In addition, its missingness-aware learning strategy is specifically designed for mosaic data configurations, allowing robust integration even when modalities are incompletely observed or unevenly distributed across batches. Together, these design features make GAMMI well suited to heterogeneous integration settings with incomplete observations and batch variation.

The resulting integrated representations were sufficiently stable and informative to support downstream analyses relevant to real biological studies, including spatial localization of major cell types, reconstruction of tissue architecture, and characterization of spatially coherent molecular patterns in the mouse cortex. Collectively, these observations indicate that GAMMI remains applicable under realistic and heterogeneous conditions; the detailed spatial integration results for this scenario are presented below.

### Spatial integration of mouse cortex

Using this composite mouse cortex scenario, we next examined the detailed integration performance of GAMMI across heterogeneous spatial batches and performed a comparative evaluation with existing methods. Single-cell RNA-seq and ATAC-seq data were obtained from Chen *et al.*, providing paired transcriptome and chromatin accessibility profiles at single-cell resolution. Spatial transcriptomics data were collected from two biologically distinct batches of mouse cortex (GSM6704282 and GSM6704280), introducing batch-specific and biological variability. This configuration creates a highly challenging integration scenario involving multiple modalities and multiple spatial batches with intrinsic biological differences.

**Figure 4:**
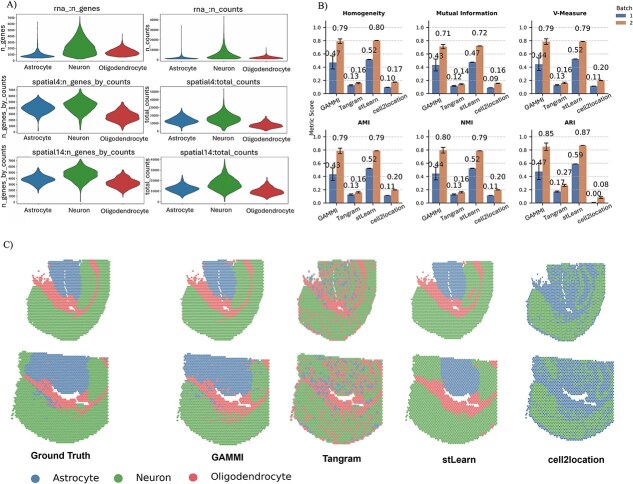
Spatial integration and cell type mapping in mouse cortex using GAMMI and benchmarking with existing methods. (a) Violin plots showing the distributions of total UMI counts, number of detected genes, and spatial marker gene expression across two spatial batches after integration. (b) Quantitative evaluation of integration performance using six clustering-based metrics (homogeneity, mutual information, V-measure, adjusted mutual information, normalized mutual information, and Adjusted Rand Index). (c) Spatial reconstruction of major cell types in the mouse cortex, comparing the ground truth with integration results from GAMMI, Tangram, stLearn, and cell2location.

Figure 4a illustrates the distributions of total unique molecular identifier (UMI) counts, the number of detected genes, and selected spatial marker gene expressions across the two spatial batches after integration. The violin plots reveal the underlying heterogeneity in the raw spatial datasets and provide a basis for assessing the integration effects across different methods. Quantitative evaluation results are shown in Fig. 4b. Six clustering-based metrics: Homogeneity, Mutual Information, V-Measure, Adjusted Mutual Information (AMI), Normalized Mutual Information (NMI), and Adjusted Rand Index (ARI), ©were computed to assess the integration quality across the two spatial batches. The contribution of spatial neighborhood edges was further assessed by ablation analysis, with batch-specific clustering results summarized in Supplementary Table S4. Among the benchmarked methods, stLearn [8] achieves slightly higher scores (~1%), whereas GAMMI delivers comparable performance and remains competitive across all metrics, demonstrating effective preservation of biologically meaningful structures. Spatial reconstruction results are presented in Fig. 4c. GAMMI successfully recapitulates the major anatomical domains, producing coherent and well-defined spatial boundaries for cell types such as astrocytes, oligodendrocytes, and excitatory neurons. While stLearn [8] attains slightly higher clustering agreement in the quantitative metrics, its reconstructed domains show more boundary diffusion in certain regions. In contrast, GAMMI maintains sharper spatial boundaries and reduced background noise compared with Tangram [7] and cell2location [6], reflecting its strength in structural preservation during spatial integration.

To further validate GAMMI’s applicability in multi-omics integration scenarios, we additionally applied the model to a paired scRNA-seq and scATAC-seq dataset from the LU927 lung cancer sample. By combining chromatin accessibility information with transcriptomic profiles, GAMMI successfully refined heterogeneous RNA clusters into biologically coherent subpopulations and revealed modality-supported lineage structures. These results highlight the strong biological interpretability of GAMMI in paired multi-omics settings, with detailed marker-gene analyses and annotated UMAP visualizations provided in Supplementary Fig. S11.

Together, these results demonstrate that GAMMI provides a robust and generalizable solution for integrating heterogeneous single-cell and spatial data by learning relational structure under realistic mosaic and batch-confounded conditions.

## Discussion

As outlined in the Introduction, a central obstacle in practical multi-omics studies is integration under mosaic and spatial designs, where missingness, limited overlap, and batch structure are tightly coupled. GAMMI was developed to directly address this setting. Across diverse mosaic single-cell benchmarks and spatial scenarios, GAMMI achieves robust cross-domain integration while preserving biologically interpretable structure, including in challenging low-overlap and fully unpaired regimes (Fig. 2). Collectively, these results reinforce a key message: in realistic mosaic settings, integration quality is often determined less by model capacity than by whether a method (i) learns biologically meaningful relational structure rather than relying on direct cell or sample alignment, (ii) respects the observational nature of missingness, and (iii) suppresses technical variation at the level where representations are formed.

Mechanistically, GAMMI’s performance advantages can be directly traced to the design principles highlighted in the Introduction and help explain why several representative multi-omics integration methods become less robust as mosaic structure intensifies. First, addressing the challenge that unobserved modalities are frequently and incorrectly treated as negatives in mosaic designs, GAMMI is explicitly missingness-aware: it distinguishes unobserved entries (unmeasured modalities) from true zeros and constructs negative supervision only when the corresponding modality is observed. This design avoids false-negative signals arising from unmeasured modalities under severe missingness and likely contributes to GAMMI’s stability in low-overlap regimes (Fig. 2). Second, responding to the difficulty of cross-modality information propagation when shared cells or modalities collapse, GAMMI remains effective by enabling anchor-free information flow through a heterogeneous graph with feature mediator nodes and feature–feature links. In contrast, approaches that rely on stronger overlap or pairing structures can become sensitive when shared cells or modalities are limited. For example, although scMoMaT [19] is explicitly designed for mosaic integration, its alignment can still depend on the availability of informative overlap and may be challenged when missingness patterns and domain shifts are severe. Similarly, methods such as GLUE [14] or MIDAS [18] leverage cross-modality structure, but their performance can degrade in highly unpaired designs when the ‘bridges’ required for propagation—such as shared anchors, reliable correspondences, or sufficiently informative feature links—become weak. Third, in settings where batch effects correlate with platform differences and missingness patterns, GAMMI performs adversarial batch removal at the embedding level. This is critical because such coupled batch effects cannot be fully resolved by preprocessing or post hoc correction alone, in contrast to batch-correction-first strategies such as Harmony [17]. Together, these mechanisms explain why GAMMI reduces modality-driven separation and fragmentation while preserving interpretable biological structure in regimes where alternative pipelines often degrade.

Beyond alignment, GAMMI reframes integration as an interpretable discovery framework by jointly embedding cells and molecular features and by treating spatial data as a structurally informative modality that constrains representation learning. This design directly addresses a limitation of correspondence-driven spatial mapping approaches, which typically rely on post hoc projection of single-cell states onto spatial coordinates. In GAMMI, molecular programs—including genes, peaks, and proteins—organize around the cell states they support, enabling direct prioritization of regulatory signals and markers without requiring separate association analyses. Importantly, feature nodes are not merely an interpretability add-on; they function as mediator entities that enable cross-modality message passing, supporting alignment and discovery even in the absence of shared cells or explicit anchors. In disease analyses, this design links robust integration to testable hypotheses by connecting disease-enriched subpopulations to pathway-level perturbations.

In spatial settings, a distinct challenge is incorporating tissue architecture as a constraint during representation learning rather than relying on post hoc spatial mapping. This is where GAMMI differs conceptually from correspondence-driven frameworks such as Tangram [7] or cell2location [6], which focus on projecting single-cell states onto spatial coordinates and may face a trade-off between spatial coherence and molecular fidelity. By encoding spatial adjacency within the same heterogeneous graph used for multi-omics learning, GAMMI leverages spatial structure to regularize integration and to enrich molecular representations across spatial locations, supporting coherent anatomical reconstruction and spatially informed marker discovery.

Several limitations and future directions remain. Graph construction requires choices of edge definitions and hyperparameters, such as spatial neighborhood scales and the relative weighting of edge types, which may require dataset-specific tuning; adaptive schemes that learn neighborhood scales or edge weights could reduce manual effort. Moreover, as with any propagation-based framework, performance will inevitably be limited when cross-modality links are extremely weak; accordingly, we stress-tested GAMMI using sparsity simulations (Supplementary Fig. S10). Finally, GAMMI learns a snapshot embedding and does not explicitly model temporal dynamics or state transitions. Extending the framework to trajectory inference and disease progression modeling represents a natural and important direction for future work.

## Method

### Overview of the GAMMI workflow

GAMMI is designed for unified integration under realistic mosaic and spatial settings, where modalities can be missing, overlaps can be partial or absent, and batch effects can be coupled with experimental design. The method consists of five components: (i) a lightweight preprocessing pipeline to preserve native data structure while ensuring comparability across modalities; (ii) heterogeneous graph construction to encode quantitative cell–feature relationships, cross-modality feature associations, and (when available) spatial structural constraints; (iii) edge-based contrastive learning that enables representation learning without cell–cell pairing, equipped with missingness-aware negative sampling to avoid false-negative supervision; (iv) adversarial batch correction at the embedding level to suppress domain-specific signals while retaining biological variation; and (v) global optimization strategies and a comprehensive evaluation protocol. We describe each component below.

### Graph construction

GAMMI represents heterogeneous multi-omics measurements as a single heterogeneous graph so that integration can be performed as structure-aware representation learning rather than relying on direct cell–cell pairing or modality-specific feature-space alignment. This graph formulation is particularly suited to mosaic and spatial settings: it provides a unified object in which information can propagate across modalities through explicit relational structure, and it naturally accommodates missing modalities, partial overlap, and spatial neighborhood constraints.

Formally, we construct a heterogeneous graph$G=\left(V,E\right)$with two types of nodes: cell nodes ${V}_c$ (cells or spatial spots) and feature nodes ${V}_f$(molecular features such as genes, peaks, proteins, or methylation loci). Let ${N}_c$ and ${N}_f$ denote the number of cell and feature nodes. Then, $\left|{V}_c\right|={N}_c\kern0.5em \left|{V}_f\right|={N}_f$, and the total number of nodes is $\left|V\right|={N}_c+{N}_f$. We assign unified indices to all nodes, where cell nodes are indexed from$0$ to ${N}_c-1$, and feature nodes are indexed from ${N}_c$ to ${N}_c+{N}_f-1$, allowing for all node embeddings to be stored in a shared representation matrix.

Edges *E* encode three complementary relation types: (i) "cell–feature" edges that preserve quantitative molecular measurements without requiring feature-space alignment, (ii) "feature–feature" edges that provide cross-modality coupling and enable information propagation even when cells are unpaired, and (iii) "spatial cell–cell" edges that incorporate tissue organization by treating spatial omics as a structural-constraint modality. We detail the construction and weighting of each edge type below.

### Cell-feature edges (quantitative molecular relations)

For each cell node ${c}_i\in{V}_c$ and feature node ${g}_j\in{V}_f$, if feature $j$ is detected in cell $i$, we create an edge between ${c}_i\kern0em$and ${g}_j$​. To improve comparability and training stability, we apply log transformation followed by min-max normalization to the raw expression values ${x}_{ij}$. The edge weight is defined as:


1 \begin{eqnarray*}& {w}_{ij}^{(cf)}=\nonumber\\ &\frac{\log \left(1+{x}_{ij}\right)-\min \left\{\log \left(1+{x}_{ij}\right):{c}_i\in{V}_c\right\}}{\max\!\, \left\{\log \left(1\,{+}\,{x}_{ij}\right):{c}_i\,{\in}\, {V}_c,{g}_j\,{\in}\, {V}_f\right\}-\min\!\, \left\{\log \left(1\,{+}\,{x}_{ij}\right): {c}_i\,{\in}\, {V}_c,{g}_j\,{\in}\, {V}_f\right\}}\qquad \nonumber\\ \end{eqnarray*}

This design preserves quantitative molecular information while avoiding explicit alignment of modality-specific feature spaces.

### Feature-feature edges (cross-modality information propagation)

A central challenge in mosaic integration is enabling cross-modality coupling when shared cells or paired measurements are limited or absent. GAMMI addresses this by introducing feature–feature edges, which encode biologically meaningful associations between molecular features using prior knowledge, thereby enabling cross-modality information propagation independently of cell-level overlap. In this study, we construct feature–feature edges for commonly encountered multi-omics pairs. For RNA–ATAC coupling, we encode putative cis-regulatory relationships by connecting each gene to ATAC peaks within a ± 150 kb window around its transcription start site, motivated by evidence that regulatory elements can act over hundreds of kilobases [e.g. Cicero (Pliner *et al.*) [20]], For RNA–ADT coupling, we encode transcription-to-protein correspondence by matching antibody targets to gene symbols using curated aliases and case-insensitive string matching, enabling integration even in the absence of shared cell barcodes. These modality-spanning associations collectively define a sparse feature–feature adjacency structure that supports cross-modality information flow during graph-based representation learning. Importantly, this construction is not limited to the modalities considered here: as long as biologically grounded feature-level associations are available or can be inferred, analogous feature–feature edges can be defined for additional omics layers.

### Spatial cell–cell edges (spatial modality as a structural constraint)

When spatial coordinates are available, GAMMI incorporates spatial omics as a structurally informative modality to capture local tissue organization. Rather than enforcing hard spatial alignment, spatial cell–cell edges are introduced as a soft structural constraint that encourages neighborhood continuity and provides complementary spatial context for multi-omics integration.

For cells or spots ${c}_i$ and ${c}_j$ with Euclidean distance ${d}_{ij}$, spatial edges are considered when the pair lies within a local neighborhood defined by a distance threshold δ. The contribution of spatial proximity is modeled using an exponentially decaying function of distance,


2 \begin{eqnarray*} {w}_{ij}^{\left(\mathrm{cc}\right)}=\alpha \cdotp \exp \left(-\frac{d_{ij}}{\sigma}\right) \end{eqnarray*}

where $\sigma$ controls the rate at which spatial influence decays with distance, and α serves as a global scaling factor that modulates the overall contribution of spatial edges relative to other edge types in the heterogeneous graph.

Importantly, these parameters are not intended to define a precise physical or biological interaction model. Instead, δ, $\sigma$, and α function as structural control parameters that regulate the range and strength of spatial regularization. Their values are specified based on the spatial resolution of the assay, the characteristic scale of local neighborhoods, and the desired level of smoothness, rather than being tuned to directly optimize downstream performance. Unless otherwise stated, we use a default spatial neighborhood threshold of δ = 40, a decay scale $\sigma$ set proportional to the empirical distribution of neighbor distances, and a global spatial scaling factor α = 1, to ensure reproducibility across experiments.

In practice, spatial edges are constructed dynamically during training and act as a soft structural prior that complements molecular- and feature-based relationships without dominating the overall integration process. With the heterogeneous graph defined, integration becomes an edge-based representation learning problem. We next introduce our contrastive learning objective and the missingness-aware negative sampling strategy tailored for mosaic settings.

### Contrastive graph embedding with missingness-aware negative sampling

With the heterogeneous graph constructed, we learn a shared embedding for all nodes so that connected cell–feature, feature–feature, and spatial cell–cell relations are preserved in the latent space. The training objective combines edge-based contrastive learning with adversarial batch correction to learn robust representations from heterogeneous multi-omics data. We construct a unified embedding matrix $E\in{R}^{\left|V\right|\times d}$, where each row $E\left[i\right]$ encodes the *d*-dimensional representation of node *i*. Training encourages nodes connected by meaningful biological or spatial edges to be close in the latent space, while unrelated node pairs are separated. After training, the shared latent space enables downstream tasks such as cell clustering, cross-modal association analysis, and regulatory inference.

### Edge scoring and contrastive objective

To operationalize this objective, we reformulate integration as an edge prediction problem on the heterogeneous graph. Observed edges (cell–feature, feature–feature, and spatial cell–cell) serve as positive supervision that explicitly defines which node pairs should be close in the learned embedding space. For a node pair ($i,j$), the edge score is computed by dot product:


3 \begin{eqnarray*} {s}_{ij}=\left\langle{z}_i,{z}_j\right\rangle ={\sum}_{k=1}^d{z}_i^{(k)}\cdotp{z}_j^{(k)} \end{eqnarray*}

We use a softmax-based contrastive loss. For a positive edge ($i,j$) with a set of type-consistent negative edges($i,{k}^{-}$), the loss is:


4 \begin{eqnarray*} {\mathcal{L}}_{\mathrm{edge}}=-\log \left(\frac{\exp \left({s}_{ij}/\tau \right)}{\exp \left({s}_{ij}/\tau \right)+{\sum}_k\exp \left({s}_{ik}^{-}/\tau \right)}\right) \end{eqnarray*}

Where $\mathrm{\tau}$ is the temperature parameter.

### Matched negative sampling under mosaic missingness

In mosaic settings, many entries are unobserved due to missing modalities rather than representing true biological absence. Treating such unobserved entries as negatives can therefore induce systematic false-negative supervision.

To address this issue, we adopt a matched negative sampling strategy that preserves edge type while explicitly accounting for missingness.

For a cell–feature edge (${c}_i$, ${g}_j$), negative samples are drawn only from features with confirmed zero expression in cell ${c}_i$; if the value is missing due to an unmeasured modality, negative sampling is disabled for this pair. For feature–feature edges (${g}_p$, ${g}_q$), unrelated features are sampled to form negatives. For spatial cell–cell edges (${c}_i$, ${c}_j$), negatives are sampled from distant cells with spatial distance exceeding a predefined threshold. By default, four negatives are sampled per positive edge, balancing discrimination and computational efficiency.

The contrastive objective learns structure-preserving embeddings defined by biological and spatial relations, but batch effects can still dominate the latent space. We next describe adversarial domain adaptation to suppress batch-associated signals at the embedding level.

### Adversarial batch correction at the embedding level

In mosaic multi-omics integration, batch effects are often entangled with experimental platforms, sequencing depth and missingness patterns. As a result, latent representations can inadvertently encode batch-specific technical signatures, which may obscure genuine biological structure. Pre- or post-processing alone is frequently insufficient to remove such coupled biases. GAMMI therefore performs batch correction during representation learning: while optimizing graph embeddings, it explicitly suppresses batch-predictive components in the latent space to improve cross-batch comparability.

Specifically, we introduce a batch discriminator $\boldsymbol{D}\left(\cdotp \right)$ on the cell/spot embeddings ${\boldsymbol{z}}_{\boldsymbol{i}}\in \boldsymbol{R}$ to predict the batch label ${y}_{\mathrm{i}}$ Adversarial learning is implemented through two opposing objectives: the discriminator is trained to distinguish batches, whereas the embedding learner is trained to produce representations from which batch identity is difficult to infer. We realize this adversarial interaction using a Gradient Reversal Layer (GRL). GRL acts as the identity function in the forward pass, but reverses the gradient from the batch-classification loss in the backward pass, thereby pushing embeddings toward batch-invariant representations.

The discriminator is optimized with a multi-class cross-entropy loss:


5 \begin{eqnarray*} {\mathcal{L}}_{\mathrm{domain}}=-{\sum}_{i=1}^N{w}_i\cdotp{\sum}_{c=1}^C{y}_i^{(c)}\mathit{\log}{p}_i^{(c)} \end{eqnarray*}

where ${p}_i^{(c)}=D{\left({z}_{\mathrm{i}}\right)}_{\mathrm{c}}$ denotes the predicted probability of batch c, ${y}_i^{(c)}$​ is the one-hot domain label, and $\boldsymbol{C}$ is the number of batches. We optimize the joint objective


6 \begin{eqnarray*} {\mathcal{L}}_{\mathrm{total}}={\mathcal{L}}_{\mathrm{edge}}+\lambda{\mathcal{L}}_{\mathrm{domain}} \end{eqnarray*}

and use GRL such that ${\mathcal{L}}_{\mathrm{domain}}$ is minimized when updating the discriminator, while inducing gradients of opposite direction when updating embeddings. In this way, ${\mathcal{L}}_{\mathrm{edge}}$​ preserves biological structure defined by cell–feature, feature–feature and spatial adjacency edges, whereas the adversarial term continuously attenuates batch-associated signals.

A key consideration is to avoid over-alignment that could remove true biological differences. We therefore treat $\lambda$ as a tunable hyperparameter and adopt early stopping based on validation loss to improve generalization. GAMMI additionally supports optional batch-specific embedding offsets to absorb global batch shifts, reducing the burden on adversarial learning and improving training stability.

Finally, to avoid conflating batch effects with structural patterns introduced by feature-level priors, we apply the adversarial constraint primarily to cell/spot node embeddings. Together with the structure-preserving contrastive objective, this design yields unified embeddings that are biologically coherent yet robust to batch effects.

### Global optimization strategy

The overall objective jointly optimizes biological relevance and domain invariance:


7 \begin{eqnarray*} {\mathcal{L}}_{\mathrm{total}}={\mathcal{L}}_{\mathrm{edge}}+\lambda{\mathcal{L}}_{\mathrm{domain}} \end{eqnarray*}

where $\lambda$ controls the strength of domain alignment.

To further account for batch-specific variability, GAMMI optionally uses batch-specific embedding offsets, in which each batch has a learnable offset vector added to base cell embeddings. We also use early stopping based on validation loss to prevent overfitting and to improve generalization.

We next describe the evaluation framework used to benchmark integration quality across datasets, batches, modalities, and spatial settings.

## Training and evaluation

### Model training and hyperparameter

GAMMI is trained by optimizing the edge-based contrastive objective on the heterogeneous graph using stochastic gradient descent. During training, node embeddings are updated to bring biologically or structurally related nodes closer in the latent space while separating unrelated pairs.

Unless otherwise stated, four negative edges are sampled for each positive edge, following a matched negative sampling strategy that preserves edge type consistency. This setting provides a practical balance between discriminative strength and computational efficiency and was found to yield stable optimization across datasets with varying degrees of sparsity and missingness.

For spatial transcriptomics data, spatial cell–cell edges are constructed based on Euclidean distance in physical space. A distance threshold δ is used to define local neighborhoods, such that spatial edges are added only between cells or spatial spots whose distance is smaller than δ. This design reflects the assumption that spatial influence is predominantly local and avoids imposing excessive smoothness across distant tissue regions. Spatial edges are incorporated during training as structural constraints within the same contrastive learning objective, allowing spatial organization to directly regularize representation learning rather than being applied post hoc.

Batch effects are addressed during training through adversarial domain adaptation at the embedding level. A batch discriminator is jointly optimized with the embedding model via a gradient reversal mechanism, encouraging the learned representations to be informative of biological structure while minimizing batch-predictive signals. The strength of adversarial alignment is controlled by a tunable hyperparameter, and early stopping is applied based on validation loss to prevent over-correction.

### Model evaluation

To comprehensively evaluate the performance of GAMMI, we designed a multi-dimensional benchmarking framework that jointly assesses biological consistency, batch effect correction, and—when applicable—spatial alignment. The selected evaluation metrics and benchmarking strategy follow established practices in single-cell and multi-omics integration studies, ensuring comparability with prior work and methodological traceability [21].

Biological consistency reflects the model’s ability to preserve cell population structure and was quantified using standard clustering-based metrics, including Isolated Labels, Leiden NMI, Leiden ARI, Silhouette Label, and cLISI. These metrics collectively evaluate the separation of biologically distinct populations and the compactness of inferred clusters.

Batch effect correction was assessed using Silhouette Batch, iLISI, and KBET, which capture both local and global batch mixing behavior. Together, these measures quantify the extent to which technical variation is suppressed without compromising biological identity. For an integrated summary, we additionally computed a composite score that aggregates biological and batch-related metrics to provide an overall assessment of integration quality.

In spatial transcriptomics scenarios, spatial alignment was evaluated using six commonly adopted clustering agreement metrics—Homogeneity, Mutual Information, V-Measure, AMI, NMI, and ARI—which quantify consistency between inferred cell-type labels and known spatial domains. UMAP visualizations were further used for qualitative assessment of spatial continuity, boundary preservation, and anatomical coherence.

This unified evaluation framework enables robust comparison of GAMMI with baseline methods across diverse datasets, integration regimes, and experimental conditions, ensuring that observed performance gains are not driven by a single metric or data setting.

### Data collection and preprocessing

To evaluate GAMMI under realistic mosaic and spatial integration settings, we curated seven representative datasets spanning single-cell, spatial, and disease scenarios, covering fully paired, partially overlapping, and completely unpaired designs. These include PBMC, MOP, BMMC, Muto, Triple, a spatial multi-omics dataset, and an Alzheimer’ s disease dataset.

Briefly, PBMC (~17 k cells) comprises four batches profiled with combinations of RNA, ATAC, and ADT, with ADT shared across batches. MOP (~77 k mouse brain cells) represents a minimal-anchor setting, where only one batch contains both RNA and ATAC. BMMC (~37 k cells) includes RNA, ATAC, and ADT across two batches, with limited anchoring strength. Muto (~44 k cells) forms a diagonal mosaic design with paired RNA–ATAC measurements but no shared modalities across batches. Triple integrates three independent mouse brain datasets measuring RNA, ATAC, and DNA methylation, respectively, with no shared cells or batches.

To assess spatial integration, we included a spatial multi-omics dataset (~18 k mouse brain spots profiled with RNA and ATAC) with spatial coordinates defining local adjacency. A single-cell RNA-seq dataset from an Alzheimer’s disease mouse model (~13 k cells) was further used to evaluate performance in a disease context.

Since GAMMI operates on graph edges rather than modality-specific reconstruction, we adopt a lightweight and unified preprocessing strategy that stabilizes numerical scale while preserving native mosaic structure. Batch labels and missingness patterns are retained and explicitly used during training. Full dataset descriptions, preprocessing details, and implementation settings are provided in Supplementary Methods.

**Key Points**

- GAMMI is a graph-guided framework for integrating single-cell and spatial multi-omics data under realistic mosaic designs with incomplete and unpaired modalities.
- GAMMI learns biologically meaningful relational structure, rather than relying on direct cell or sample alignment, by jointly embedding cells and molecular features.
- A missingness-aware contrastive objective avoids false-negative supervision, enabling stable integration in low-overlap regimes.
- Spatial transcriptomics is incorporated as a learning-time structural constraint rather than through post hoc mapping.
- GAMMI consistently outperforms existing methods in biological conservation, batch correction and spatial reconstruction across diverse datasets.

## Supplementary Material

bbag218_Supplementary

## Data Availability

All datasets analyzed in this study were obtained from publicly accessible repositories. The paired scRNA-seq and scATAC-seq LU927 dataset was downloaded from the NCBI Gene Expression Omnibus (GEO) under accession number GSE255330, and the Alzheimer’s disease dataset from GSE138852. Additional multi-omics datasets, including BMMC, PBMC, MUTO, MOP, and TRIPLE, were retrieved from publicly released resources associated with their original publications. The ZT4 and ZT14 spatial transcriptomics datasets (GSM6704280 and GSM6704282) were obtained from GEO. A complete list of links to all data sources is available at: https://github.com/luyiyun/graph_mosaic_integration. No new experimental datasets were generated in this study.

## Code availability

The code to reproduce the results in this manuscript is available at https://github.com/luyiyun/graph_mosaic_integration.

## References

[1] Tang F, Barbacioru C, Wang Y et al. mRNA-Seq whole-transcriptome analysis of a single cell. *Nat Methods* 2009;6:377–82. 10.1038/nmeth.1315 19349980

[2] Stuart T, Satija R. Integrative single-cell analysis. *Nat Rev Genet* 2019;20:257–72. 10.1038/s41576-019-0093-7 30696980

[3] Hasin Y, Seldin M, Lusis A. Multi-omics approaches to disease. *Genome Biol* 2017;18:83. 10.1186/s13059-017-1215-1 28476144 PMC5418815

[4] Ståhl PL, Salmén F, Vickovic S et al. Visualization and analysis of gene expression in tissue sections by spatial transcriptomics. *Science.* 2016;353:78–82. 10.1126/science.aaf2403 27365449

[5] Argelaguet R, Cuomo ASE, Stegle O et al. Computational principles and challenges in single-cell data integration. *Nat Biotechnol* 2021;39:1202–15. 10.1038/s41587-021-00895-7 33941931

[6] Kleshchevnikov V, Shmatko A, Dann E et al. Cell2location maps fine-grained cell types in spatial transcriptomics. *Nat Biotechnol* 2022;40:661–71. 10.1038/s41587-021-01139-4 35027729

[7] Biancalani T, Scalia G, Buffoni L et al. Deep learning and alignment of spatially resolved single-cell transcriptomes with tangram. *Nat Methods* 2021;18:1352–62. 10.1038/s41592-021-01264-7 34711971 PMC8566243

[8] Pham D, Tan X, Balderson B et al. Robust mapping of spatiotemporal trajectories and cell–cell interactions in healthy and diseased tissues. *Nat Commun* 2023;14:7739. 10.1038/s41467-023-43120-6 38007580 PMC10676408

[9] Argelaguet R, Arnol D, Bredikhin D et al. MOFA+: a statistical framework for comprehensive integration of multi-modal single-cell data. *Genome Biol* 2020;21:111. 10.1186/s13059-020-02015-1 32393329 PMC7212577

[10] Demetci P, Santorella R, Sandstede B et al. SCOT: single-cell multi-omics alignment with optimal transport. *J Comput Biol* 2022;29:3–18. 10.1089/cmb.2021.0446 35050714 PMC8812493

[11] Hao Y, Hao S, Andersen-Nissen E et al. Integrated analysis of multimodal single-cell data. *Cell.* 2021;184:3573–87.e29. 10.1016/j.cell.2021.04.048 34062119 PMC8238499

[12] Lopez R, Regier J, Cole MB et al. Deep generative modeling for single-cell transcriptomics. *Nat Methods* 2018;15:1053–8. 10.1038/s41592-018-0229-2 30504886 PMC6289068

[13] Welch JD, Kozareva V, Ferreira A et al. Single-cell multi-omic integration compares and contrasts features of brain cell identity. *Cell.* 2019;177:1873–87.e17. 10.1016/j.cell.2019.05.006 31178122 PMC6716797

[14] Cao Z-J, Gao G. Multi-omics single-cell data integration and regulatory inference with graph-linked embedding. *Nat Biotechnol* 2022;40:1458–66. 10.1038/s41587-022-01284-4 35501393 PMC9546775

[15] Chen H, Ryu J, Vinyard ME et al. SIMBA: single-cell embedding along with features. *Nat Methods* 2024;21:1003–13. 10.1038/s41592-023-01899-8 37248389 PMC11166568

[16] Wang J, Ma A, Chang Y et al. scGNN is a novel graph neural network framework for single-cell RNA-Seq analyses. *Nat Commun* 2021;12:1882. 10.1038/s41467-021-22197-x 33767197 PMC7994447

[17] Korsunsky I, Millard N, Fan J et al. Fast, sensitive and accurate integration of single-cell data with harmony. *Nat Methods* 2019;16:1289–96. 10.1038/s41592-019-0619-0 31740819 PMC6884693

[18] He Z, Hu S, Chen Y et al. Mosaic integration and knowledge transfer of single-cell multimodal data with MIDAS. *Nat Biotechnol* 2024;42:1594–605. 10.1038/s41587-023-02040-y 38263515 PMC11471558

[19] Zhang Z, Sun H, Mariappan R et al. scMoMaT jointly performs single cell mosaic integration and multi-modal bio-marker detection. *Nat Commun* 2023;14:384. 10.1038/s41467-023-36066-2 36693837 PMC9873790

[20] Pliner HA, Packer JS, McFaline-Figueroa JL et al. Cicero predicts cis-regulatory DNA interactions from single-cell chromatin accessibility data. *Mol Cell* 2018;71:858–71.e8. 10.1016/j.molcel.2018.06.044 30078726 PMC6582963

[21] Xiao C, Chen Y, Meng Q et al. Benchmarking multi-omics integration algorithms across single-cell RNA and ATAC data. *Brief Bioinform* 2024;25:bbae095. 10.1093/bib/bbae095 38493343 PMC10944570

