# Supplementary material for: GAMMI: graph-guided contrastive and adversarial integration of single-cell and spatial multi-omics data
Source: Brief Bioinform. 2026 May 11;27(3):bbag218. doi: 10.1093/bib/bbag218 (PMC13158126; doi:10.1093/bib/bbag218)
Supplement: bbag218_Supplementary [file bbag218_supplementary.docx]

**Data Collection**

To evaluate the robustness and generalizability of GAMMI across diverse integration scenarios, we curated seven representative datasets spanning single-cell, spatial, and disease settings: PBMC, MOP, BMMC, Muto, Triple, a spatial multi-omics dataset, and an Alzheimer’s disease dataset. These datasets cover a wide range of integration regimes, including fully shared modalities, partially overlapping modalities, and completely unpaired mosaic configurations across batches, providing stringent testbeds under realistic conditions.

The PBMC dataset comprises ~17,000 human peripheral blood mononuclear cells collected across four batches, profiled with combinations of scRNA-seq (RNA), scATAC-seq (ATAC), and ADT (209 surface markers). ADT is shared across all batches and serves as a consistent anchor. The MOP dataset contains ~77,000 mouse brain cells from five batches, with only one batch containing both RNA and ATAC and the remaining batches containing a single modality, representing a minimal-anchor setting. The BMMC dataset includes ~37,000 human bone marrow mononuclear cells from two batches profiled with RNA, ATAC, and ADT; although ADT is shared, its limited feature dimensionality restricts its anchoring strength. The Muto dataset consists of ~44,000 mouse brain cells from five batches with paired RNA and ATAC measurements, but no modality is shared across batches, forming a diagonal integration scenario in which both cells and modalities are disjoint. The Triple dataset integrates three independently generated mouse brain datasets measuring RNA, ATAC, and DNA methylation, respectively, with no overlapping batches or cells, representing an extremely heterogeneous and fully unpaired configuration.

To assess spatial applicability, we included a spatial multi-omics dataset comprising ~18,000 spatial spots from two mouse brain slices collected at Zeitgeber time points ZT4 and ZT14. Each spot is profiled with RNA and ATAC (~3,000 features per modality), and spatial coordinates are used to define spatial adjacency. Finally, to evaluate performance in a real disease setting, we included a single-cell RNA-seq dataset from an Alzheimer’s disease mouse model (13,214 hippocampal and cortical cells across control and disease conditions; ~10,850 genes).

### **Preprocessing**

Because the core learning objective in GAMMI is defined on graph edges rather than on modality-specific reconstruction, we adopt a lightweight yet unified preprocessing pipeline that stabilizes numerical scale while preserving the native mosaic structure. RNA and methylation data are log-normalized and reduced by principal component analysis (PCA), whereas sparse ATAC data are transformed by TF–IDF followed by latent semantic indexing (LSI). To reduce the influence of extreme values and to ensure consistent scaling across modalities, we further apply log and min–max normalization when constructing quantitative edge weights (see below). For datasets with shared cells (e.g., PBMC), modalities are aligned using common cell barcodes. For datasets without shared cells (e.g., Muto, Triple, and the spatial dataset), modalities are treated independently, and cross-omics feature relationships are derived from existing biological resources to support biologically informed cross-modality coupling during graph construction. Feature selection is guided by variance and graph connectivity to retain informative variables. Importantly, batch labels are preserved and explicitly used during training (rather than being removed during preprocessing), enabling embedding-level batch correction.

After preprocessing, all datasets are represented in a comparable form while retaining missingness patterns and batch structure. We next describe how GAMMI encodes these signals into a unified heterogeneous graph.


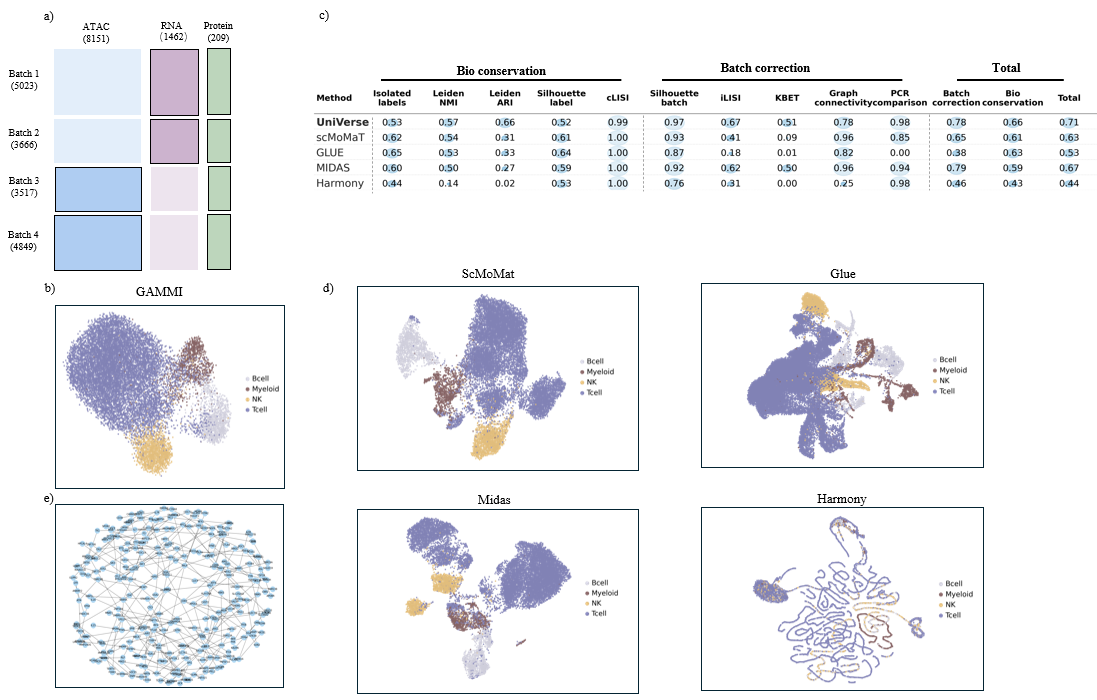


**Supplementary Figure S1 | Integration results of the PBMC dataset across GAMMI and baseline methods.**

**a**, Modality availability matrix for the PBMC dataset. This dataset comprises four batches (total 17,106 cells), with measurements from ATAC, RNA, and Protein (ADT) modalities. Protein features are shared across all batches, providing an anchor for integration.
**b**, UMAP embedding of GAMMI-integrated cells colored by cell type. GAMMI achieves clear biological separation across B cells, myeloid cells, NK cells, and T cells.
**c**, Quantitative benchmarking of GAMMI and four baseline methods (scMoMaT, GLUE, MIDAS, Harmony) using 11 metrics. GAMMI achieves the highest total integration score (0.71), with strong performance in both biological conservation and batch correction.
**d**, UMAP embeddings from baseline methods. scMoMaT and MIDAS preserve some biological structures but exhibit batch fragmentation. GLUE shows partial modality mixing, while Harmony fails to align modalities, producing overcorrected embedding.
**e**, Visualization of the feature-feature graph used in GAMMI for RNA–ADT and ATAC–RNA connectivity. Edges represent prior biological relationships incorporated into the heterogeneous graph.


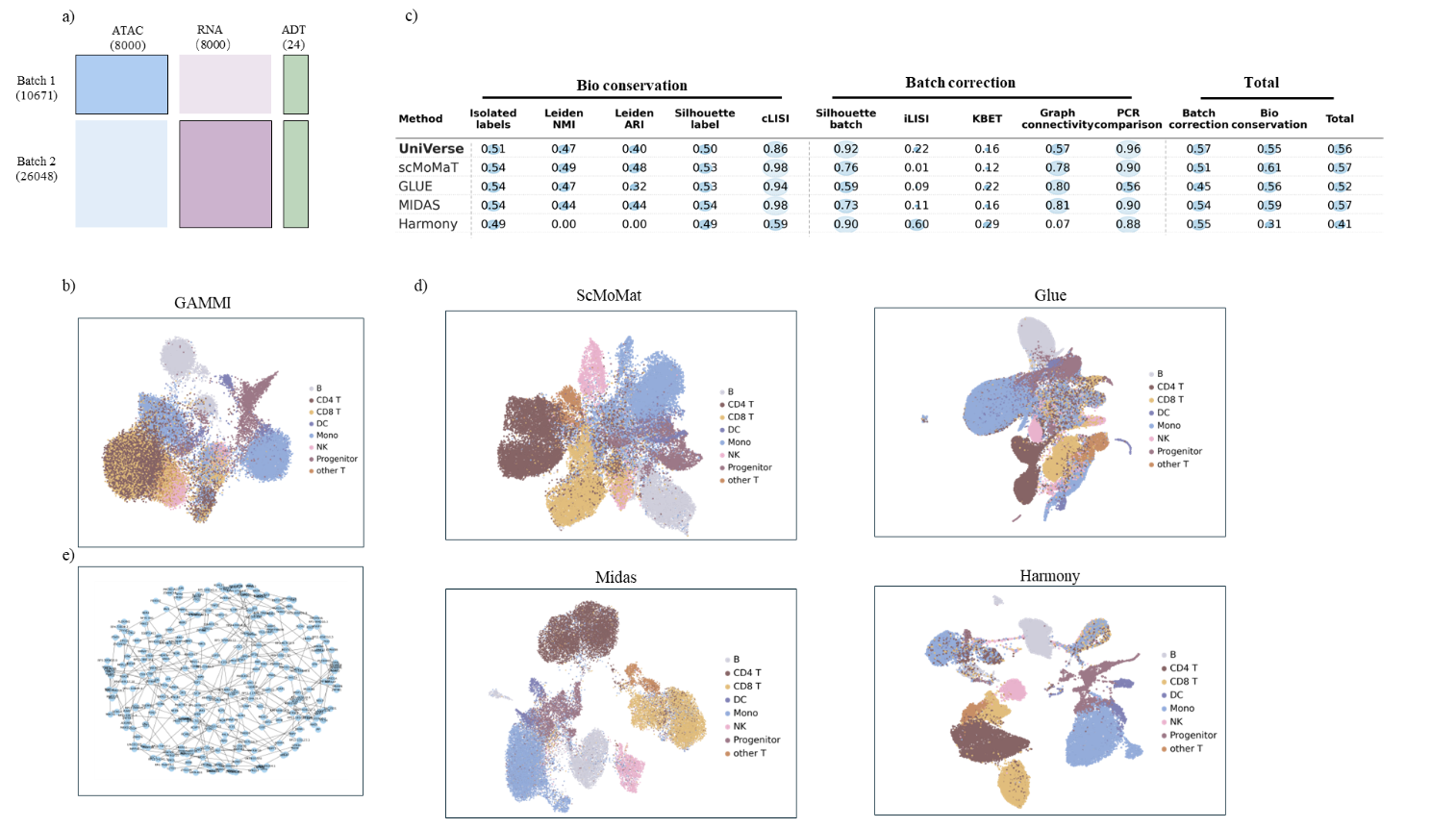


**Supplementary Figure S2 | Integration results of the BMMC dataset across GAMMI and baseline methods.**

a, Modality availability diagram for the BMMC dataset. Two batches (total 37,000+ cells) are profiled with RNA, ATAC, and protein (ADT) modalities. Only the ADT modality is shared across batches, and it contains limited features (24 markers), posing a low-information anchor challenge.
b, UMAP embedding of GAMMI integration results colored by annotated cell types. GAMMI effectively aligns batches and separates biological populations despite the weak cross-batch anchor.
c, Benchmarking results comparing GAMMI against scMoMaT, GLUE, MIDAS, and Harmony using 11 metrics covering biological conservation and batch correction. GAMMI achieves the highest total integration score (0.65), outperforming baselines in both dimensions.
d, UMAP visualizations from baseline methods. scMoMaT partially preserves cell identity but exhibits batch separation. GLUE and Harmony show modality-driven artifacts. MIDAS produces less cohesive clusters and limited batch alignment.
e, Feature-feature interaction graph for RNA–ADT and ATAC–RNA constructed based on antibody–gene alias mapping and proximity-based chromatin–gene associations. This graph provides prior structure for multimodal alignment in GAMMI.


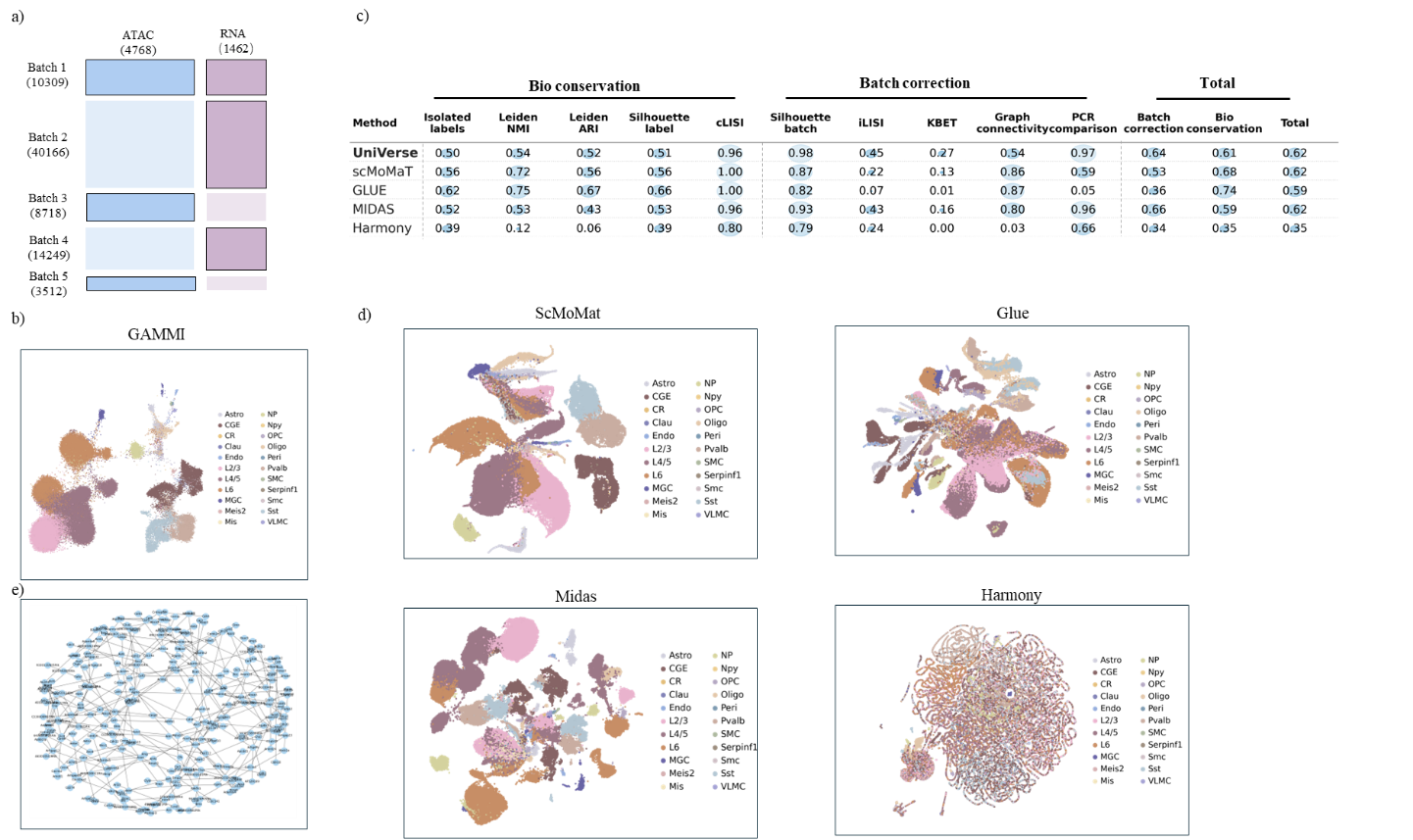


**Supplementary Figure S3 | Integration results of the MOP dataset across GAMMI and baseline methods.**

**a**, Modality distribution across five batches in the MOP dataset (total ~77,000 mouse brain cells). Only one batch includes both RNA and ATAC modalities, while the remaining batches each contain a single modality, forming a minimal-anchor scenario with no consistent overlap across batches.
**b**, UMAP embedding of GAMMI-integrated cells colored by cell type. GAMMI successfully integrates unpaired modalities and batches, preserving distinct biological clusters.
**c**, Quantitative benchmarking of GAMMI versus scMoMaT, GLUE, MIDAS, and Harmony. GAMMI achieves the highest total score (0.60), with strong performance in both biological conservation and batch mixing under minimal shared information.
**d**, UMAP results of baseline methods. scMoMaT and GLUE produce fragmented or modality-driven clusters; MIDAS partially aligns batches but with loss of resolution. Harmony fails to merge modalities, yielding overclustered structures dominated by technical effects.
**e**, Feature-feature graph used in GAMMI, incorporating ATAC–RNA regulatory associations via genomic proximity. This prior supports cross-modality alignment in the absence of shared cells.


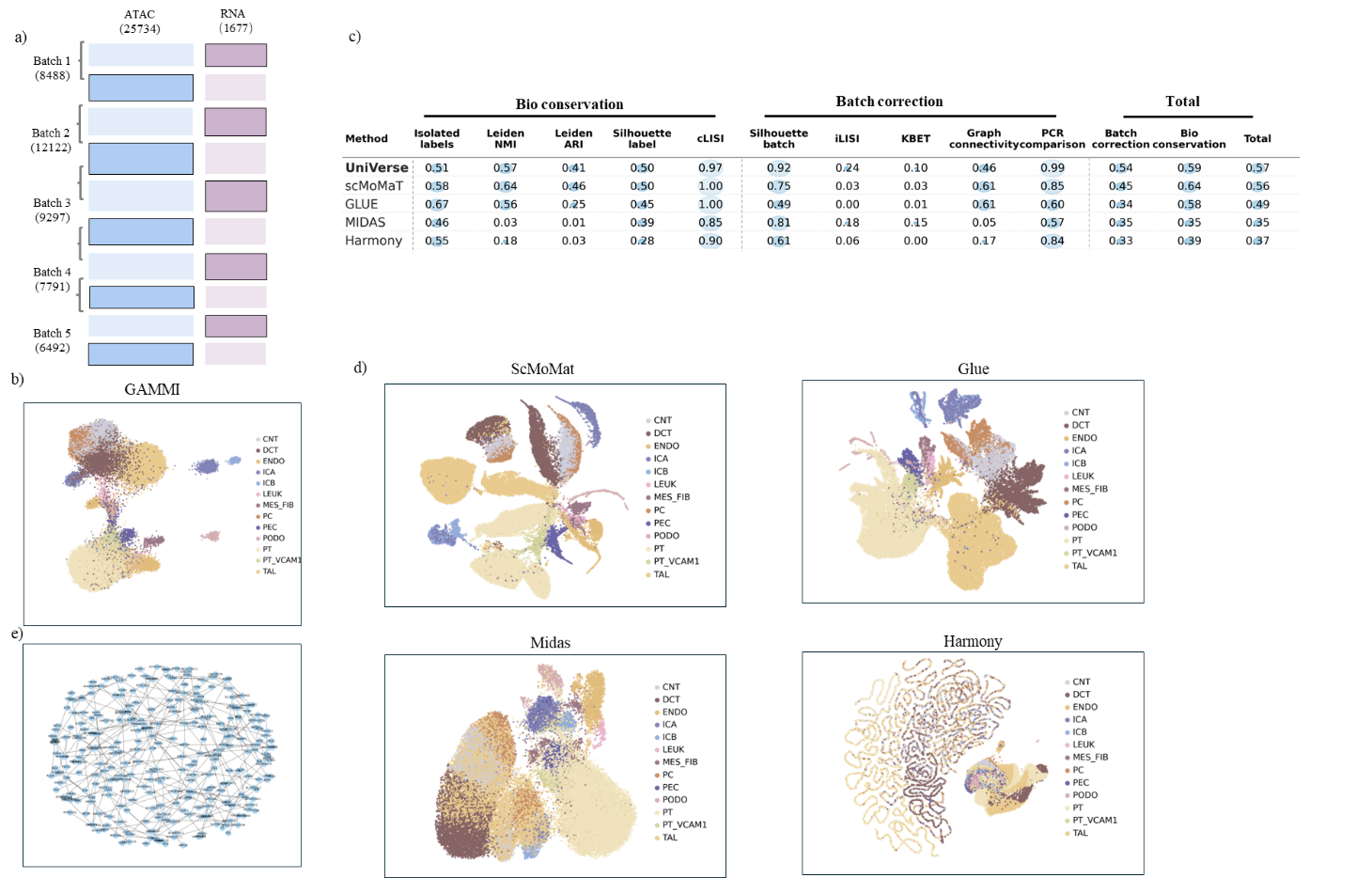


**Supplementary Figure S4 | Integration results of the Muto dataset across GAMMI and baseline methods.**

**a**, Modality distribution in the Muto dataset. Five batches (~44,000 cells total) each contain paired RNA and ATAC profiles, but no modality is shared across batches, forming a diagonal integration scenario where both cells and modalities are fully unpaired.
**b**, UMAP embedding of GAMMI integration results colored by cell type. GAMMI successfully integrates across disjoint batches and modalities, yielding coherent biological manifolds.
**c**, Quantitative comparison of GAMMI with scMoMaT, GLUE, MIDAS, and Harmony using 11 evaluation metrics. GAMMI achieves the highest total integration score (0.66), demonstrating superior robustness in fully unpaired mosaic settings.
**d**, UMAP visualizations of baseline methods. scMoMaT partially recovers biological structure but exhibits modality separation. GLUE and Harmony show overcorrection and distorted embeddings. MIDAS produces fragmented structures with loss of cell type resolution.
**e**, Feature-feature graph constructed for RNA–ATAC alignment using gene–peak proximity priors. This graph enables GAMMI to learn cross-modality associations in the absence of shared cells or anchors.


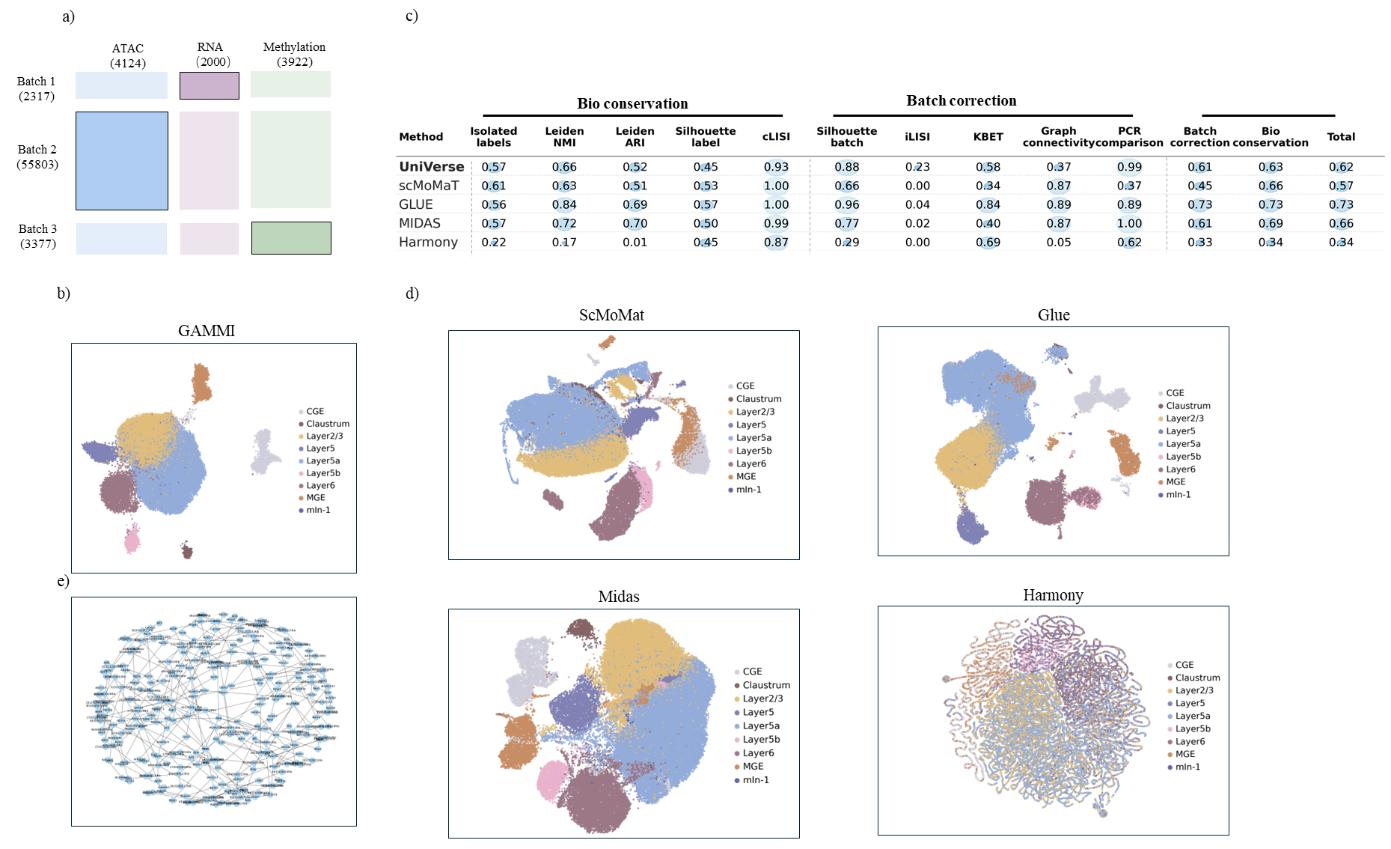


**Supplementary Figure S5 | Integration results of the Triple dataset across GAMMI and baseline methods.**

**a**, Modality distribution across three batches in the Triple dataset, each containing a distinct omics modality—RNA, ATAC, or DNA methylation. The dataset is fully unpaired in both cells and features, forming an extreme diagonal integration scenario with no overlapping batch or modality pairs.
**b**, UMAP embedding of GAMMI-integrated cells colored by cell type. GAMMI successfully bridges the three disconnected modalities, recovering coherent manifolds and preserving biologically meaningful clustering.
**c**, Quantitative benchmarking results across 11 metrics. GAMMI outperforms all baseline methods with the highest total integration score (0.63), demonstrating its superior capacity to align highly heterogeneous and unpaired data.
**d**, UMAP visualizations from scMoMaT, GLUE, MIDAS, and Harmony. All baseline methods fail to unify the three modalities: Harmony shows severe overcorrection, GLUE and scMoMaT yield modality-separated clusters, and MIDAS produces dispersed and inconsistent embeddings.
**e**, Feature-feature graph used in GAMMI for cross-modal linking of RNA, ATAC, and DNA methylation features. Edges represent biologically inferred interactions enabling the model to construct a unified embedding space despite lack of shared samples.


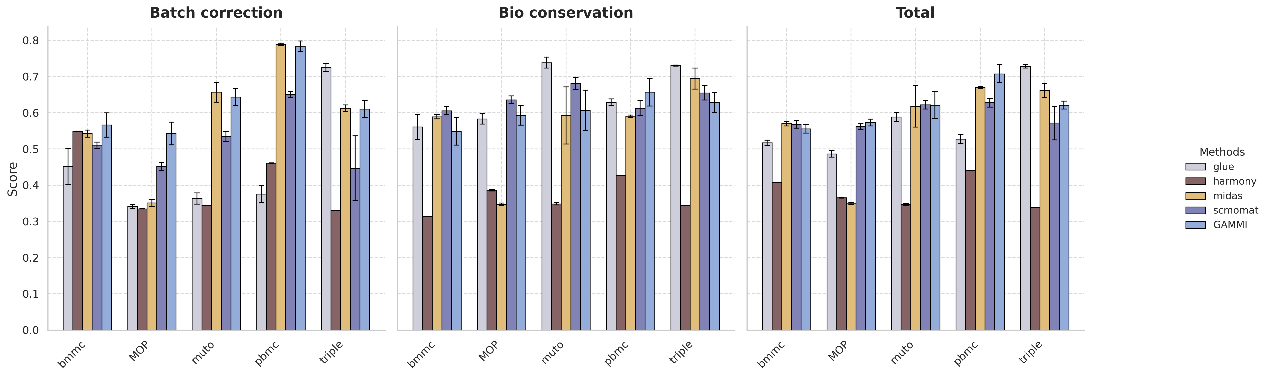


**Supplementary Figure S6 | Quantitative comparison of integration methods across five mosaic single-cell datasets.**

Bar plots summarizing the performance of five integration methods—Harmony, MIDAS, GLUE, scMoMaT, and GAMMI—on five benchmark datasets (PBMC, MOP, BMMC, Muto, and Triple) across three evaluation categories: **Batch correction** (left), **Biological conservation** (middle), and **Total score** (right).
Each bar represents the average of multiple submetrics within its category. GAMMI consistently achieves the highest or near-highest scores across all datasets and dimensions, particularly outperforming other methods in minimal-anchor (MOP) and diagonal (Muto and Triple) scenarios, demonstrating its robustness to unpaired and heterogeneous input structures.


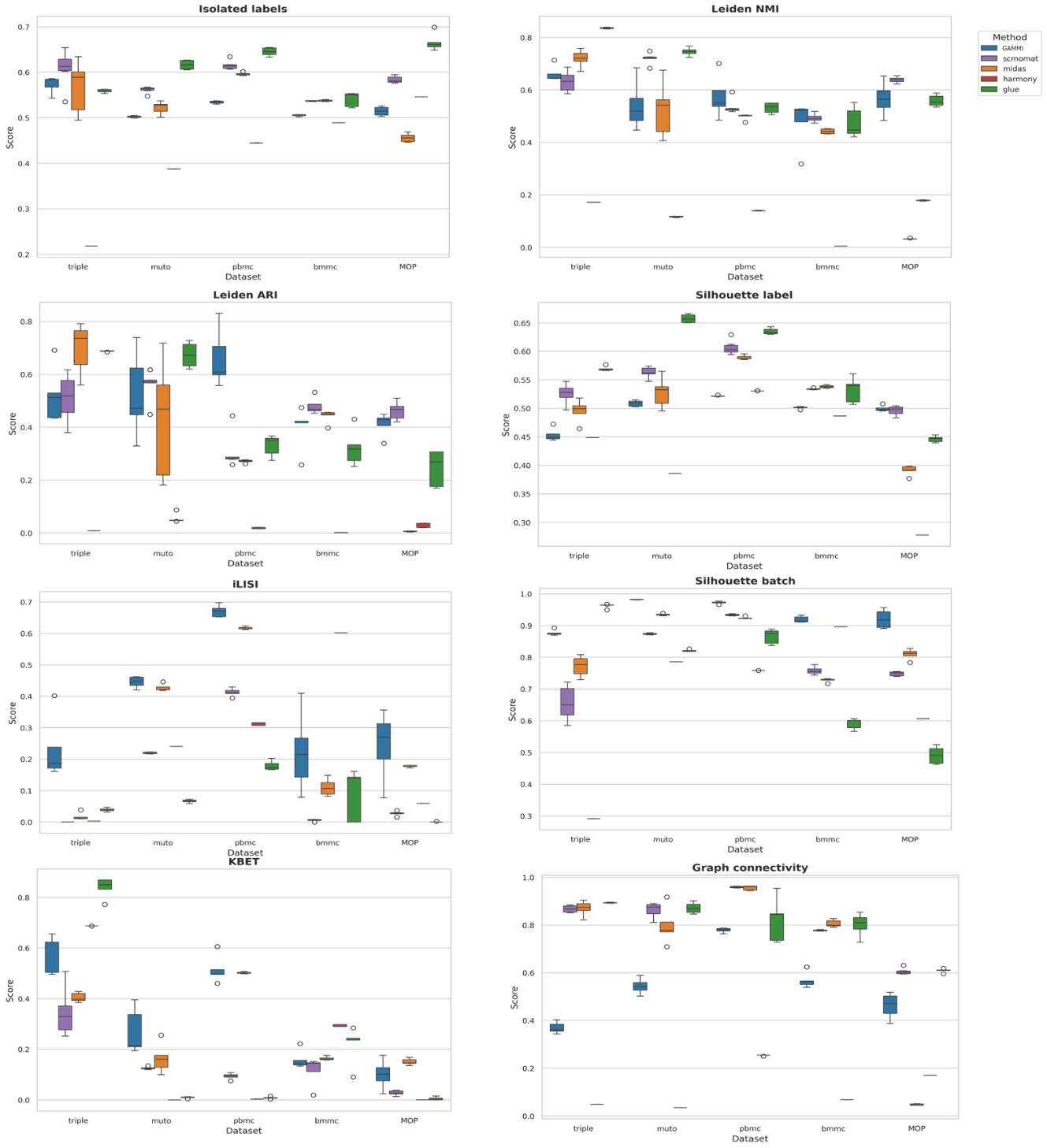


**Supplementary Figure S7 | Detailed benchmarking of integration metrics across five mosaic single-cell datasets.**

Box plots showing scores of five integration methods (GAMMI, scMoMaT, GLUE, MIDAS, Harmony) across five datasets (PBMC, MOP, BMMC, Muto, Triple) for each of the 11 evaluation metrics.
Metrics are grouped into:
**Biological conservation** — Isolated labels, Leiden NMI, Leiden ARI, Silhouette label, cLISI;
**Batch effect removal** — Silhouette batch, iLISI, KBET, Graph connectivity, PCR comparison.
GAMMI demonstrates consistently superior or comparable performance across most metrics, particularly excelling in the most challenging settings (e.g., Muto, Triple), where baselines tend to exhibit high variance or collapse. These results confirm GAMMI’s robustness and generalizability across diverse mosaic configurations and integration challenges.


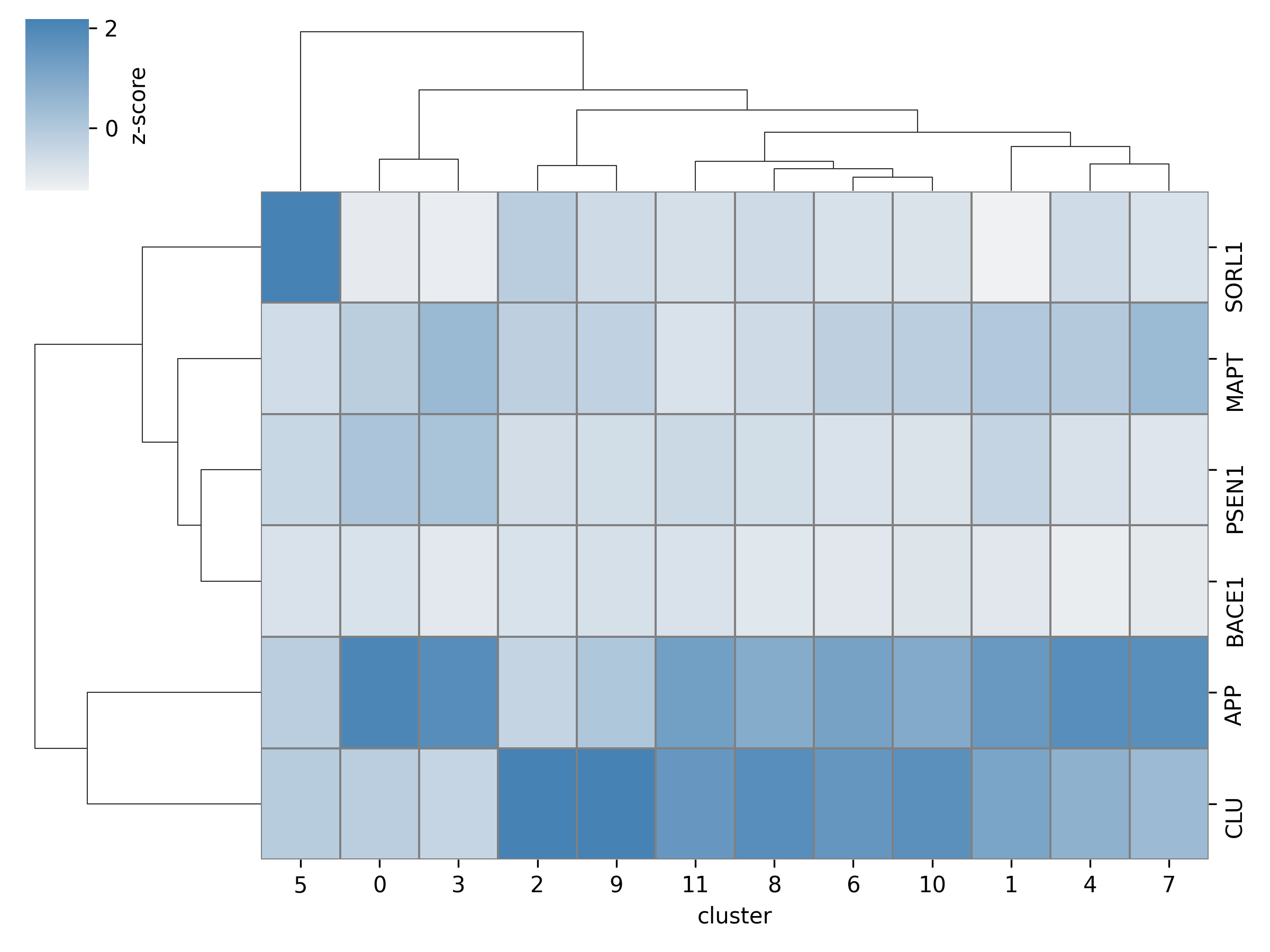


**Supplementary Figure S8 | Expression heatmap of Alzheimer's disease risk genes across GAMMI-derived clusters.**

Heatmap showing the z-score normalized expression of six well-established Alzheimer’s disease (AD) risk genes—**APP, CLU, BACE1, PSEN1, MAPT**, and **SORL1**—across 12 cell clusters identified from the GSE138852 single-nucleus RNA-seq dataset using GAMMI.
Clusters 2, 5, and 9 exhibit prominent upregulation of multiple AD-related genes, including strong co-expression of **APP**, **BACE1**, and **CLU**, suggesting potential cell states associated with amyloidogenic processing and neuroinflammatory pathways. Hierarchical clustering of genes and clusters highlights coordinated regulation and supports the utility of GAMMI in identifying disease-relevant transcriptional modules.


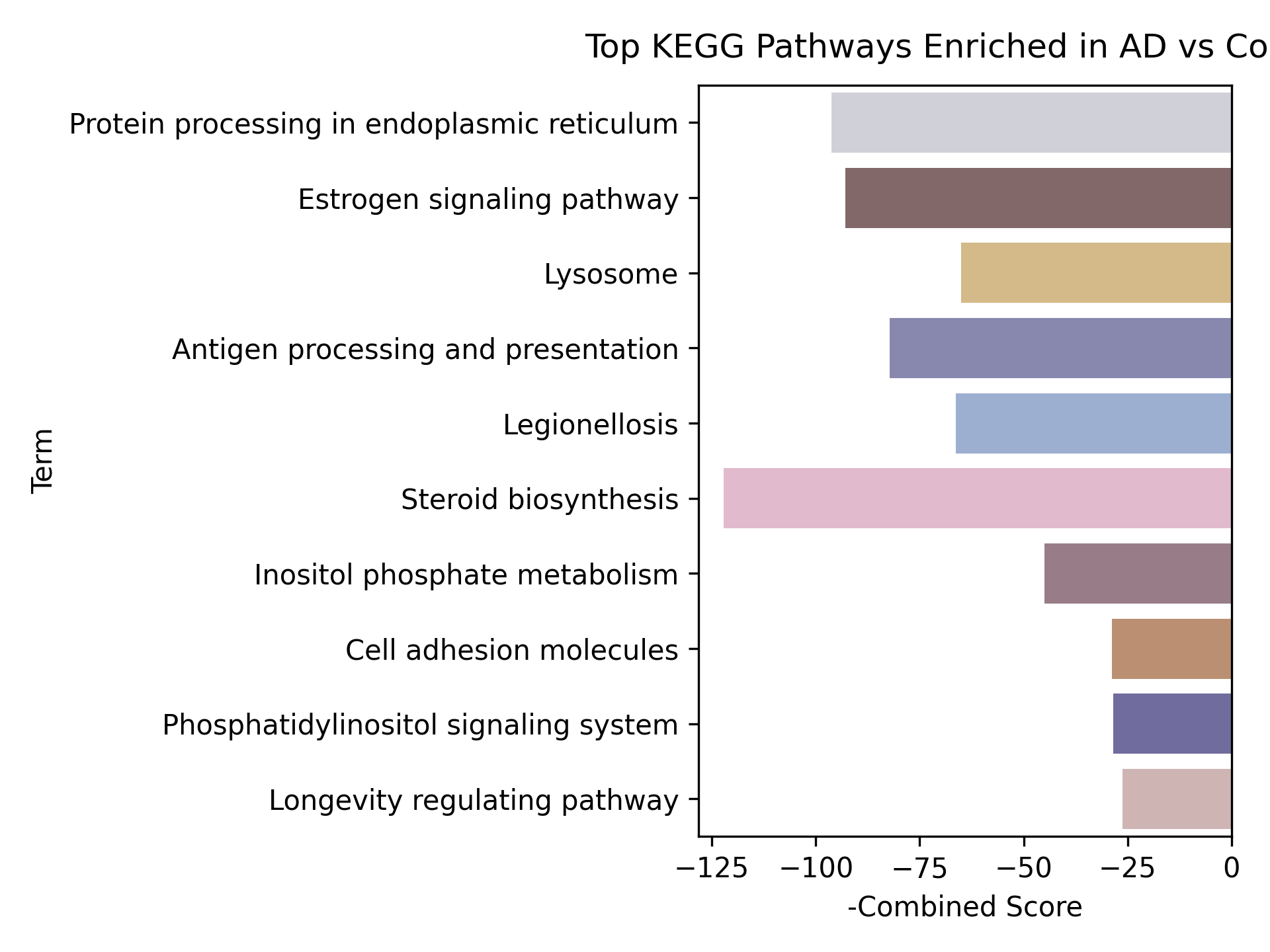


**Supplementary Figure S9 | KEGG pathway enrichment analysis of differentially expressed genes in Alzheimer’s disease.**

Bar plot showing the top 10 KEGG pathways enriched in Alzheimer's disease (AD) versus control samples based on GAMMI-derived clusters from the GSE138852 dataset.
Pathways are ranked by negative combined score, with higher values indicating stronger enrichment in AD-associated cell populations. Notably, enrichment is observed in neurodegeneration-related processes, including endoplasmic reticulum stress, lysosomal function, and steroid biosynthesis, as well as immune-related pathways such as antigen processing, cell adhesion, and phosphatidylinositol signaling. These findings highlight molecular programs implicated in AD pathogenesis and underscore the interpretability of GAMMI embeddings for functional profiling.


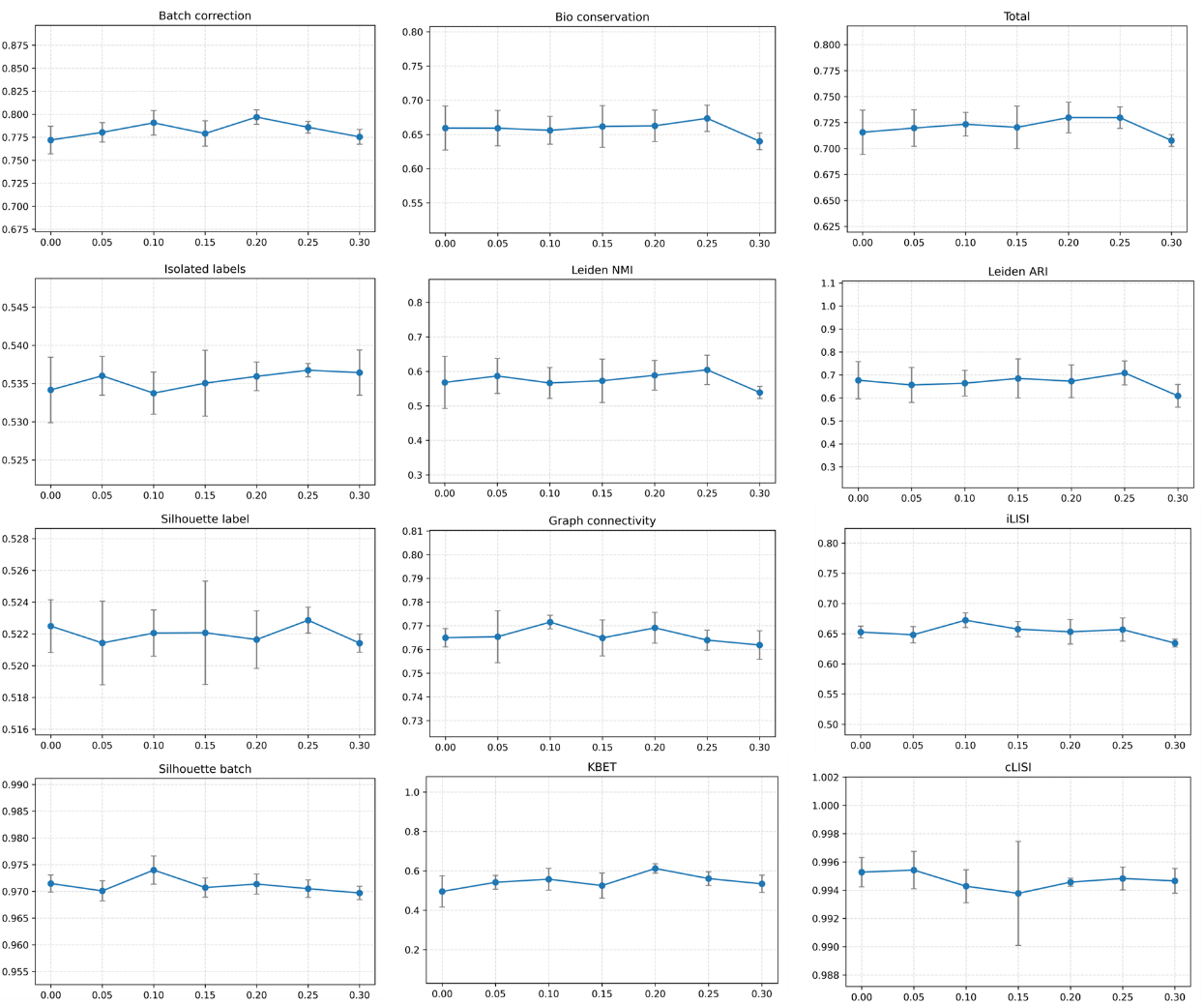


**Supplementary Figure S10｜Performance of GAMMI under increasing data sparsity.**
Each panel shows the mean (line) and standard deviation (error bars) of the corresponding evaluation metric across different sparsity levels, obtained by randomly masking entries in the input data matrices. All metrics remain largely stable as data sparsity increases from 0 to 0.3, indicating that GAMMI is robust to moderate levels of missing or corrupted values.

**Supplementary Table S1 |** Ablation analysis of key GAMMI components and hyperparameters.

| ablation | baseline | Low Learning  Rate | Low Neg  sampling | No Gradient  Reverse | No Grad Rev+ Low Neg | No Grad Rev+ No Loss cls | No cls | No cls+ Low Neg |
| --- | --- | --- | --- | --- | --- | --- | --- | --- |
| Isolated labels | 0.53 ± 0.0 | 0.5 ± 0.0 | 0.52 ± 0.0 | 0.52 ± 0.0 | 0.53 ± 0.01 | 0.52 ± 0.0 | 0.52 ± 0.0 | 0.53 ± 0.0 |
| Leiden NMI | 0.57 ± 0.08 | 0.03 ± 0.01 | 0.4 ± 0.03 | 0.59 ± 0.08 | 0.54 ± 0.02 | 0.56 ± 0.09 | 0.55 ± 0.08 | 0.53 ± 0.03 |
| Leiden ARI | 0.68 ± 0.09 | 0.03 ± 0.02 | 0.46 ± 0.07 | 0.67 ± 0.11 | 0.57 ± 0.01 | 0.68 ± 0.09 | 0.66 ± 0.1 | 0.55 ± 0.04 |
| Silhouette label | 0.52 ± 0.0 | 0.5 ± 0.0 | 0.51 ± 0.0 | 0.51 ± 0.0 | 0.52 ± 0.0 | 0.51 ± 0.0 | 0.51 ± 0.0 | 0.52 ± 0.0 |
| cLISI | 1.0 ± 0.0 | 0.86 ± 0.0 | 0.96 ± 0.01 | 0.99 ± 0.0 | 0.98 ± 0.02 | 0.99 ± 0.0 | 0.99 ± 0.0 | 0.98 ± 0.01 |
| Silhouette batch | 0.97 ± 0.0 | 0.99 ± 0.0 | 0.98 ± 0.0 | 0.92 ± 0.01 | 0.95 ± 0.01 | 0.92 ± 0.01 | 0.92 ± 0.01 | 0.96 ± 0.0 |
| iLISI | 0.65 ± 0.01 | 0.74 ± 0.01 | 0.69 ± 0.02 | 0.29 ± 0.0 | 0.33 ± 0.01 | 0.29 ± 0.0 | 0.29 ± 0.0 | 0.34 ± 0.01 |
| KBET | 0.5 ± 0.09 | 0.66 ± 0.05 | 0.44 ± 0.15 | 0.18 ± 0.03 | 0.09 ± 0.04 | 0.18 ± 0.04 | 0.17 ± 0.04 | 0.11 ± 0.05 |
| Graph connectivity | 0.76 ± 0.0 | 0.29 ± 0.01 | 0.7 ± 0.04 | 0.45 ± 0.01 | 0.74 ± 0.03 | 0.45 ± 0.01 | 0.45 ± 0.01 | 0.78 ± 0.02 |
| PCR comparison | 0.98 ± 0.01 | 0.99 ± 0.0 | 0.98 ± 0.01 | 0.72 ± 0.04 | 0.83 ± 0.01 | 0.73 ± 0.04 | 0.72 ± 0.04 | 0.83 ± 0.04 |
| Batch correction | 0.77 ± 0.02 | 0.73 ± 0.01 | 0.76 ± 0.03 | 0.51 ± 0.01 | 0.59 ± 0.01 | 0.51 ± 0.02 | 0.51 ± 0.02 | 0.6 ± 0.01 |
| Bio conservation | 0.66 ± 0.04 | 0.38 ± 0.01 | 0.57 ± 0.02 | 0.66 ± 0.04 | 0.63 ± 0.01 | 0.65 ± 0.04 | 0.65 ± 0.03 | 0.62 ± 0.02 |
| Total | 0.72 ± 0.02 | 0.56 ± 0.0 | 0.66 ± 0.02 | 0.58 ± 0.02 | 0.61 ± 0.01 | 0.58 ± 0.02 | 0.58 ± 0.02 | 0.61 ± 0.01 |

**
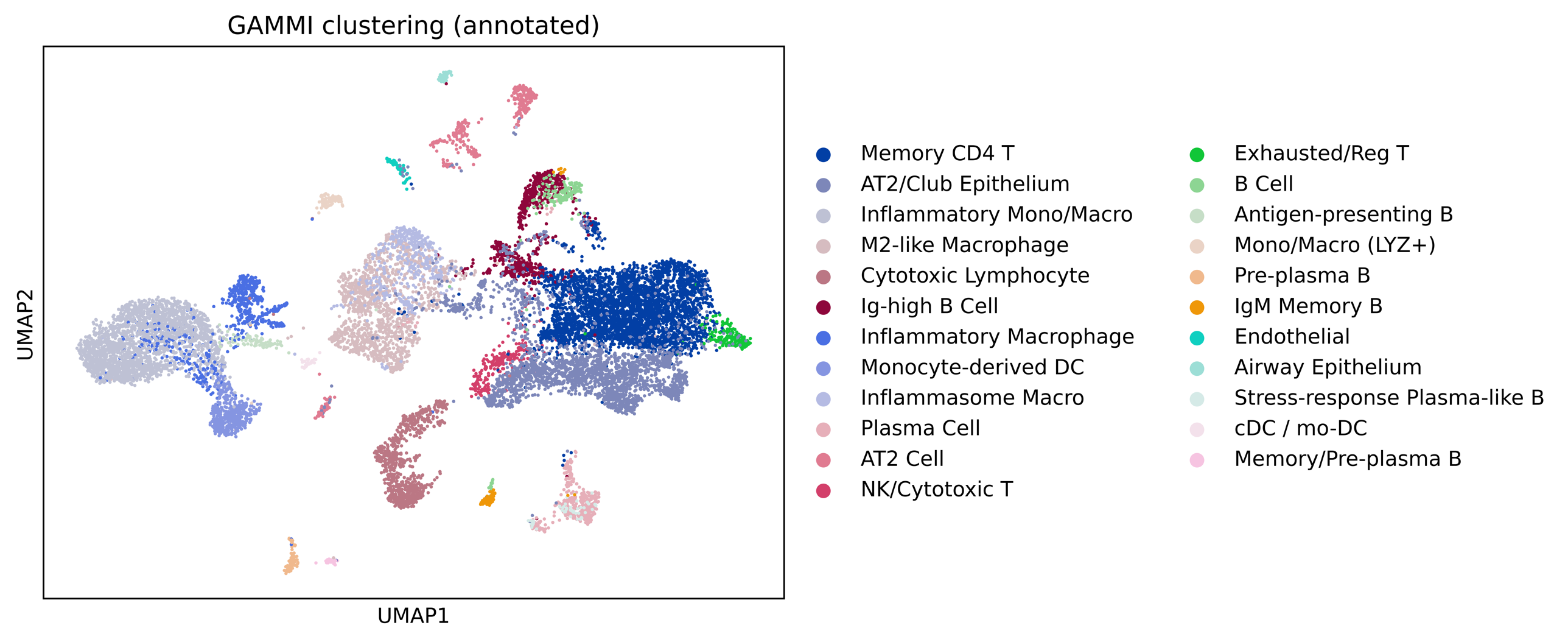
**

**Supplementary Figure S11 | UMAP visualization of GAMMI clustering with cell-type annotations.** UMAP embedding of the LU927 dataset shows GAMMI-derived clusters with refined immune and epithelial cell-type labels, including T-cell subsets, macrophage/monocyte populations, B-cell differentiation states, and epithelial lineages.


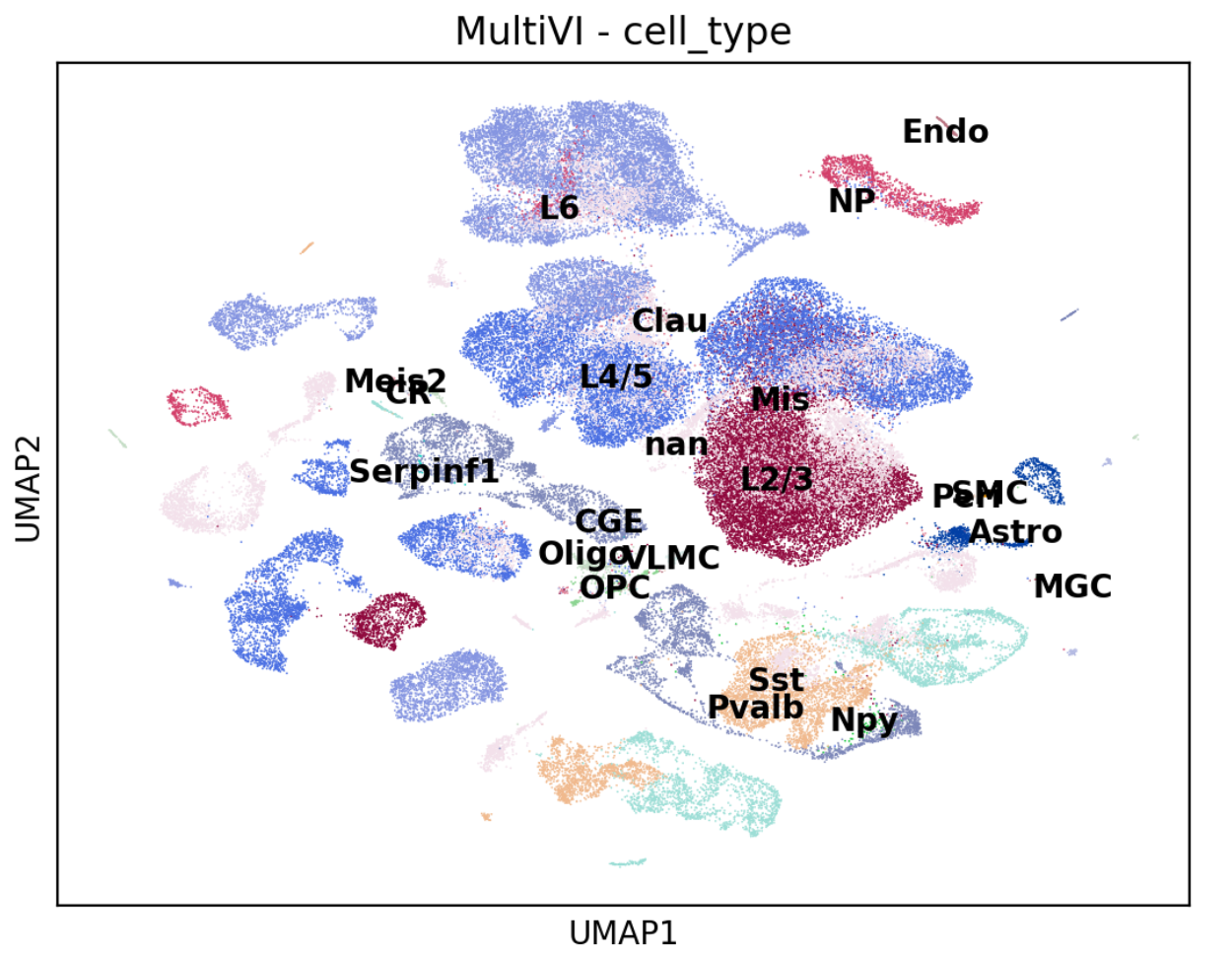


**Supplementary Figure S12 | MultiVI embedding and cell-type visualization on the MOP dataset.**

UMAP representation of the MOP dataset after MultiVI integration, colored by annotated cell types. Major neuronal, glial, and interneuron populations are clearly separated, showing the cross-modal embedding structure learned by MultiVI.


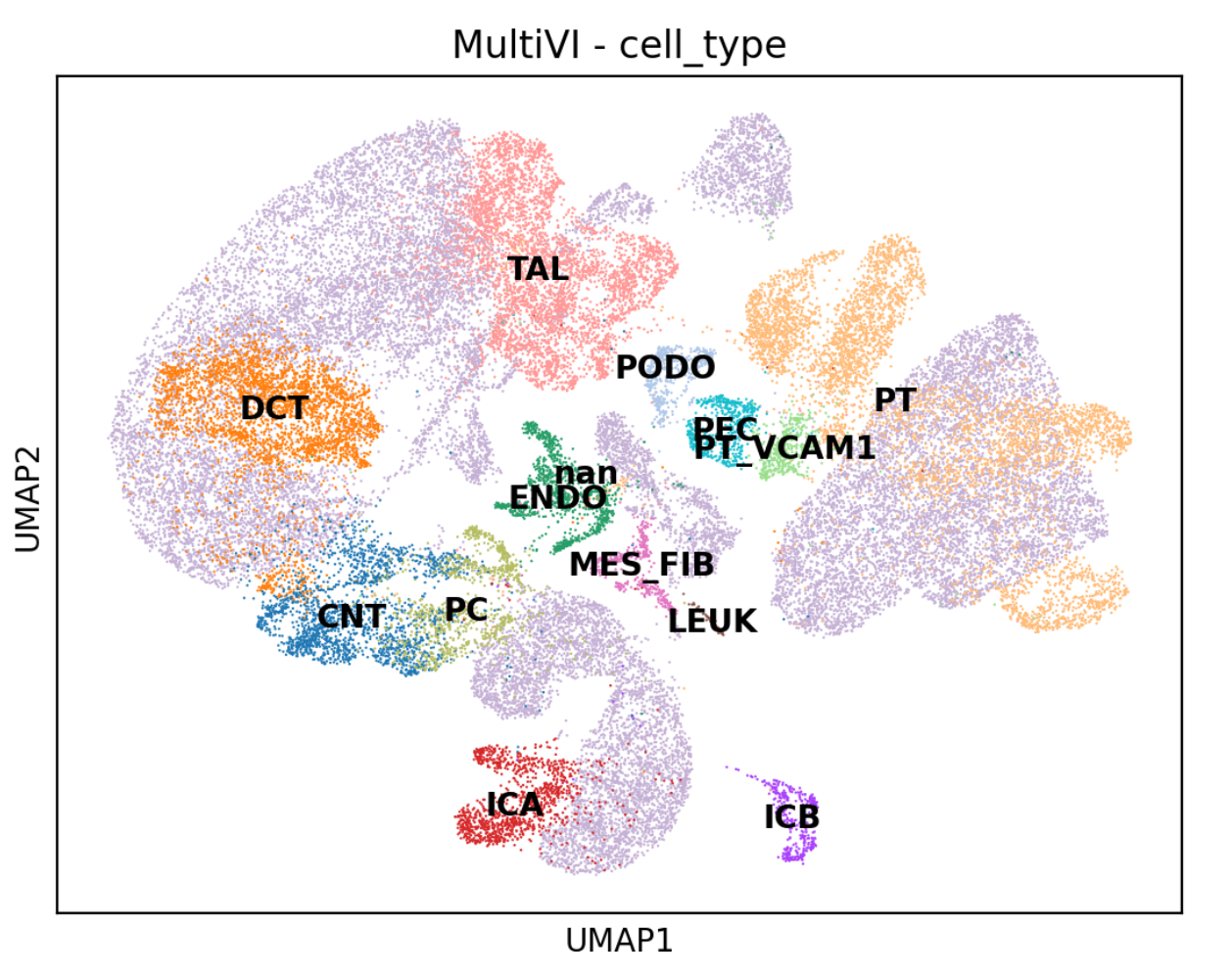


**Supplementary Figure S13 | MultiVI embedding and cell-type visualization on the MUTO dataset.**

UMAP visualization of the MUTO dataset integrated by MultiVI, with cells colored by known epithelial, stromal, endothelial, and immune cell types. MultiVI recovers major kidney cell populations and their spatially coherent manifold structure.

**Supplementary Table S2** | Benchmark metrics of MultiVI on the MOP dataset.

| Metrics | MultiVI | Metric Type |
| --- | --- | --- |
| Isolated labels | 0.67 | Bio conservation |
| KMeans NMI | 0.49 | Bio conservation |
| KMeans ARI | 0.26 | Bio conservation |
| Silhouette label | 0.48 | Bio conservation |
| cLISI | 1.00 | Bio conservation |
| Silhouette batch | 0.68 | Batch correction |
| iLISI | 0.01 | Batch correction |
| KBET | 0.06 | Batch correction |
| Graph connectivity | 0.71 | Batch correction |
| PCR comparison | 0.70 | Batch correction |
| Batch correction | 0.43 | Aggregate score |
| Bio conservation | 0.58 | Aggregate score |
| Total | 0.52 | Aggregate score |

**Supplementary Table S3** | Benchmark metrics of MultiVI on the MUTO dataset.

| Metrics | multivi | Metric Type |
| --- | --- | --- |
| Isolated labels | 0.35 | Bio conservation |
| KMeans NMI | 0.40 | Bio conservation |
| KMeans ARI | 0.07 | Bio conservation |
| Silhouette label | 0.47 | Bio conservation |
| cLISI | 1.00 | Bio conservation |
| Silhouette batch | 0.80 | Batch correction |
| iLISI | 0.00 | Batch correction |
| KBET | 0.09 | Batch correction |
| Graph connectivity | 0.96 | Batch correction |
| PCR comparison | 0.72 | Batch correction |
| Batch correction | 0.52 | Aggregate score |
| Bio conservation | 0.46 | Aggregate score |
| Total | 0.48 | Aggregate score |

**Supplementary Table S4** | Ablation of spatial neighborhood edges in SpatialGMI (clustering performance by batch).

| Metric | No spatial module  Batch1 Batch2 | | Baseline  Batch1 Batch2 | |
| --- | --- | --- | --- | --- |
| Homogeneity | 0.48 | 0.75 | 0.47 | 0.79 |
| Mutual Information | 0.44 | 0.68 | 0.43 | 0.71 |
| V-Measure | 0.44 | 0.73 | 0.44 | 0.78 |
| AMI | 0.44 | 0.73 | 0.44 | 0.78 |
| NMI | 0.44 | 0.73 | 0.44 | 0.78 |
| ARI | 0.48 | 0.78 | 0.47 | 0.85 |

**Supplementary** **Table S5 |** Statistical comparison of cell-type and subcluster proportions between AD and control samples (Wilcoxon rank-sum test).

| Cell type / subcluster | n (AD) | n (Control) | Mean AD proportion | Mean Control proportion | | p value | Significance |
| --- | --- | --- | --- | --- | --- | --- | --- |
| oligo2 | 6 | 6 | 0.0471 | 0.1884 | 0.4942 | | ns |
| oligo3 | 6 | 6 | 0.161 | 0.0072 | 0.4217 | | ns |
| astrocyte | 6 | 6 | 0.039 | 0.1216 | 0.4942 | | ns |
| oligo1 | 6 | 6 | 0.1366 | 0.0015 | 1 | | ns |
| OPC | 6 | 6 | 0.0219 | 0.0684 | 0.6081 | | ns |
| microglia | 6 | 6 | 0.0212 | 0.0238 | 0.8643 | | ns |
| doublet | 6 | 6 | 0.0243 | 0.0082 | 0.9289 | | ns |
| neuron | 6 | 6 | 0.0107 | 0.0254 | 0.4217 | | ns |
| oligo4 | 6 | 6 | 0.0004 | 0.0501 | 0.9241 | | ns |
| uniD | 6 | 6 | 0.0186 | 0 | 0.1757 | | ns |
| uniD | 6 | 6 | 0.0136 | 0 | 0.1757 | | ns |
| endo | 6 | 6 | 0.0056 | 0.0055 | 0.8643 | | ns |


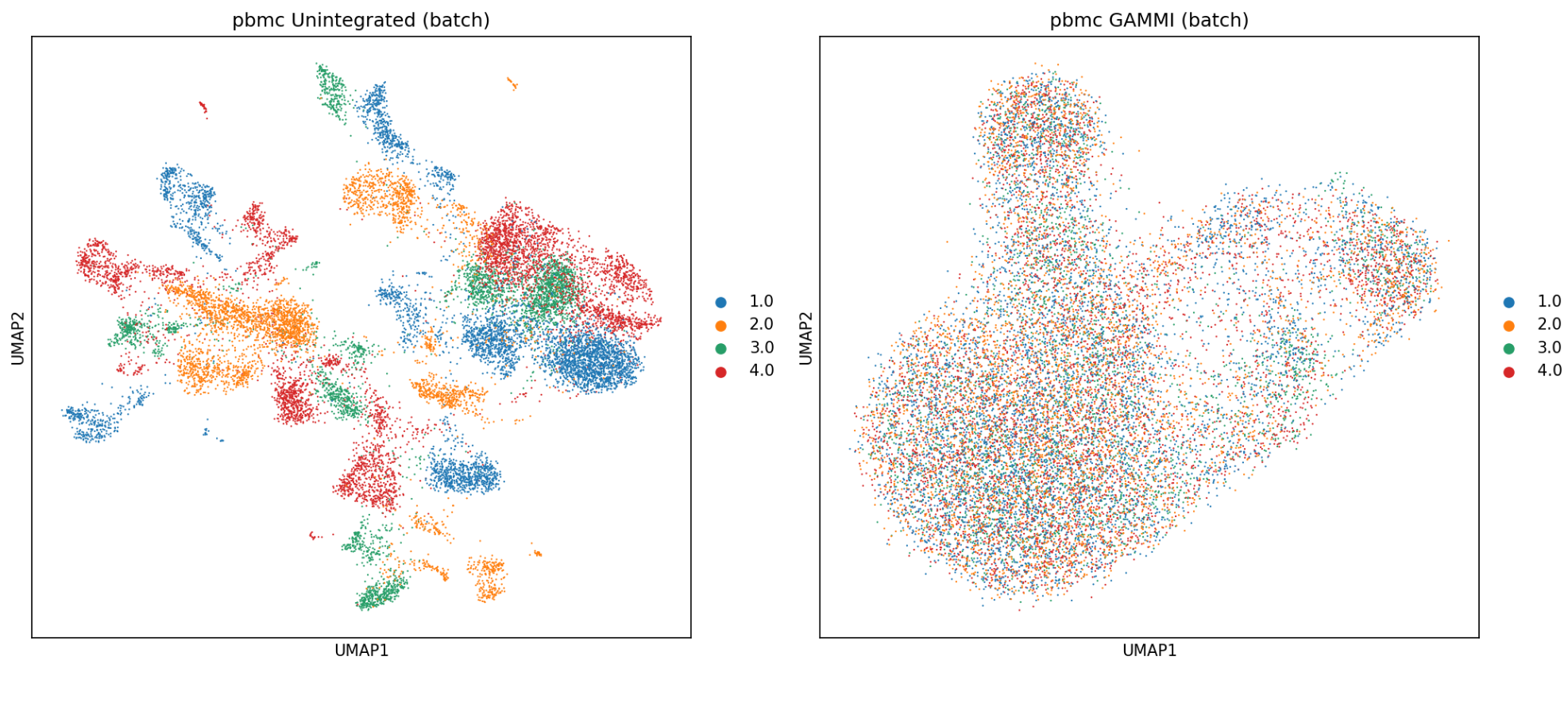


**Supplementary Figure S14 | PBMC batch correction before and after GAMMI integration.**

GAMMI markedly reduces batch separation in PBMC, producing a well-mixed embedding compared with the unintegrated data.


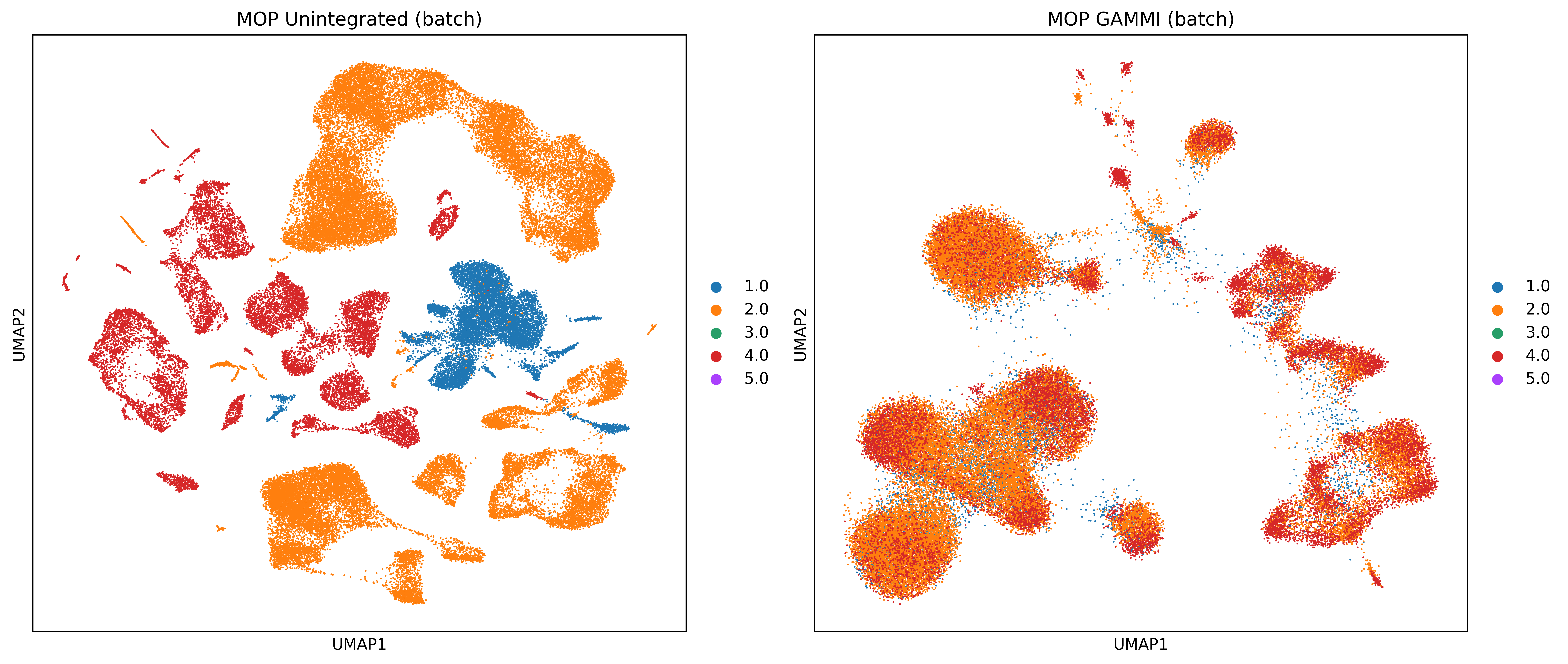


**Supplementary Figure S15 | MOP batch correction before and after GAMMI integration.**

GAMMI improves batch mixing in the MOP dataset, eliminating the strong batch-driven clusters present before integration.


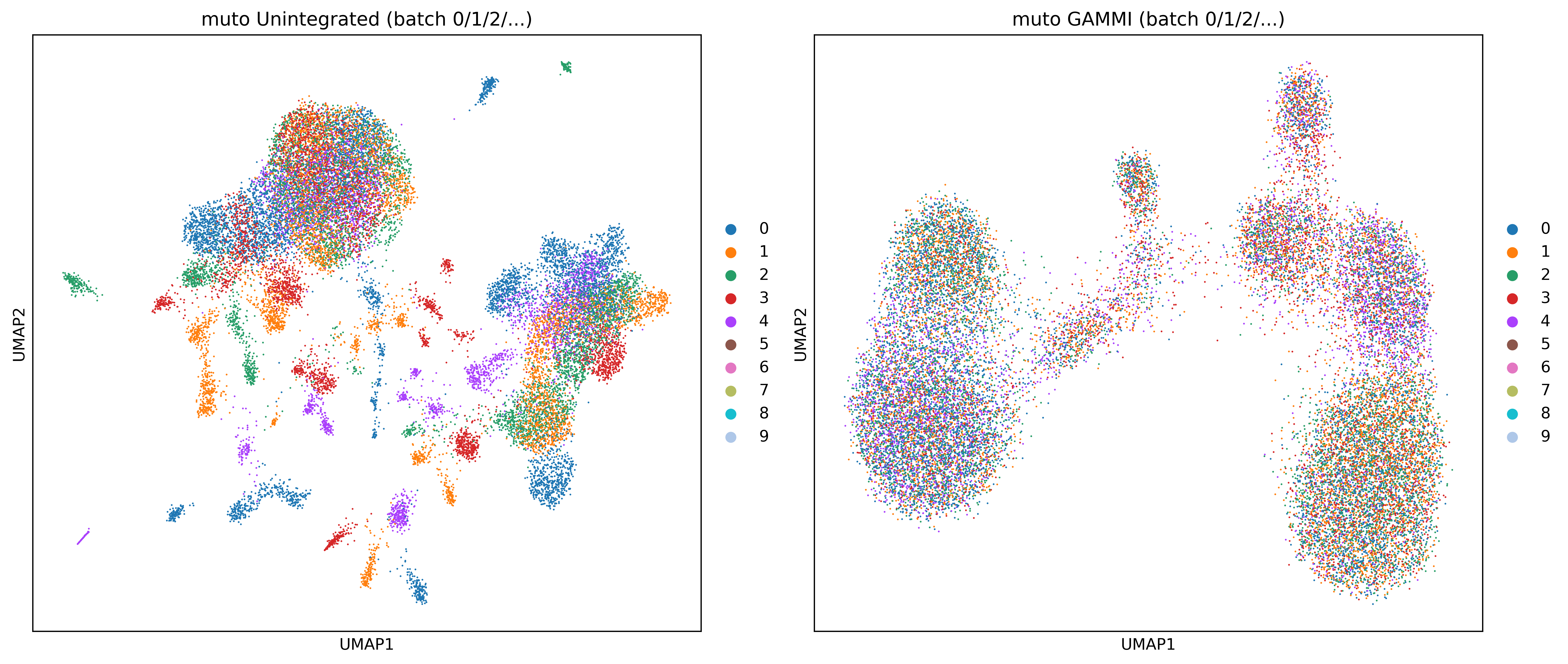


**Supplementary Figure S16 | MUTO batch correction before and after GAMMI integration.**

GAMMI integrates the heterogeneous MUTO batches into a coherent manifold, overcoming the fragmented pre-integration structure.


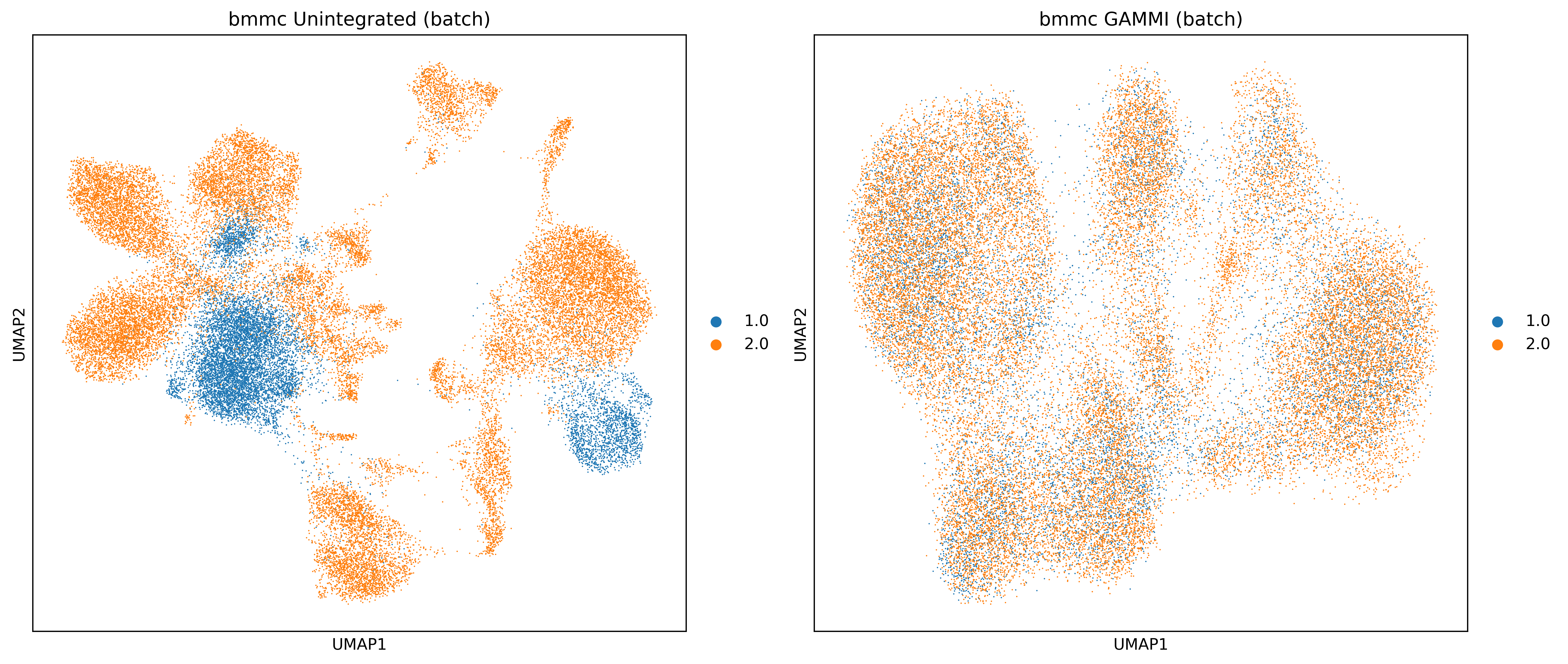


**Supplementary Figure S17 | BMMC batch correction before and after GAMMI integration.**

GAMMI effectively removes the clear batch split in BMMC, resulting in a unified embedding across batches.


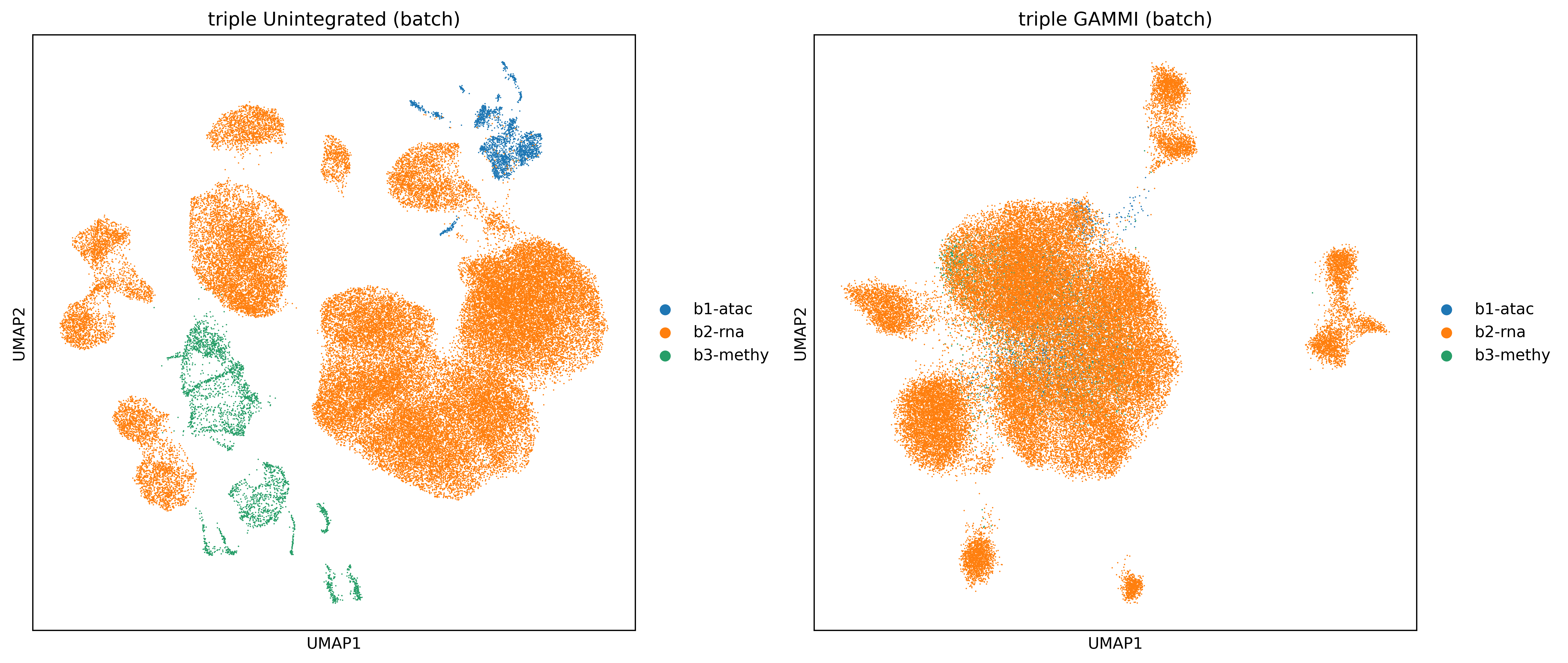


**Supplementary Figure S18 | TRIPLE batch correction before and after GAMMI integration.**

GAMMI achieves strong cross-modality mixing in the TRIPLE dataset, substantially reducing the modality separation observed before integration.


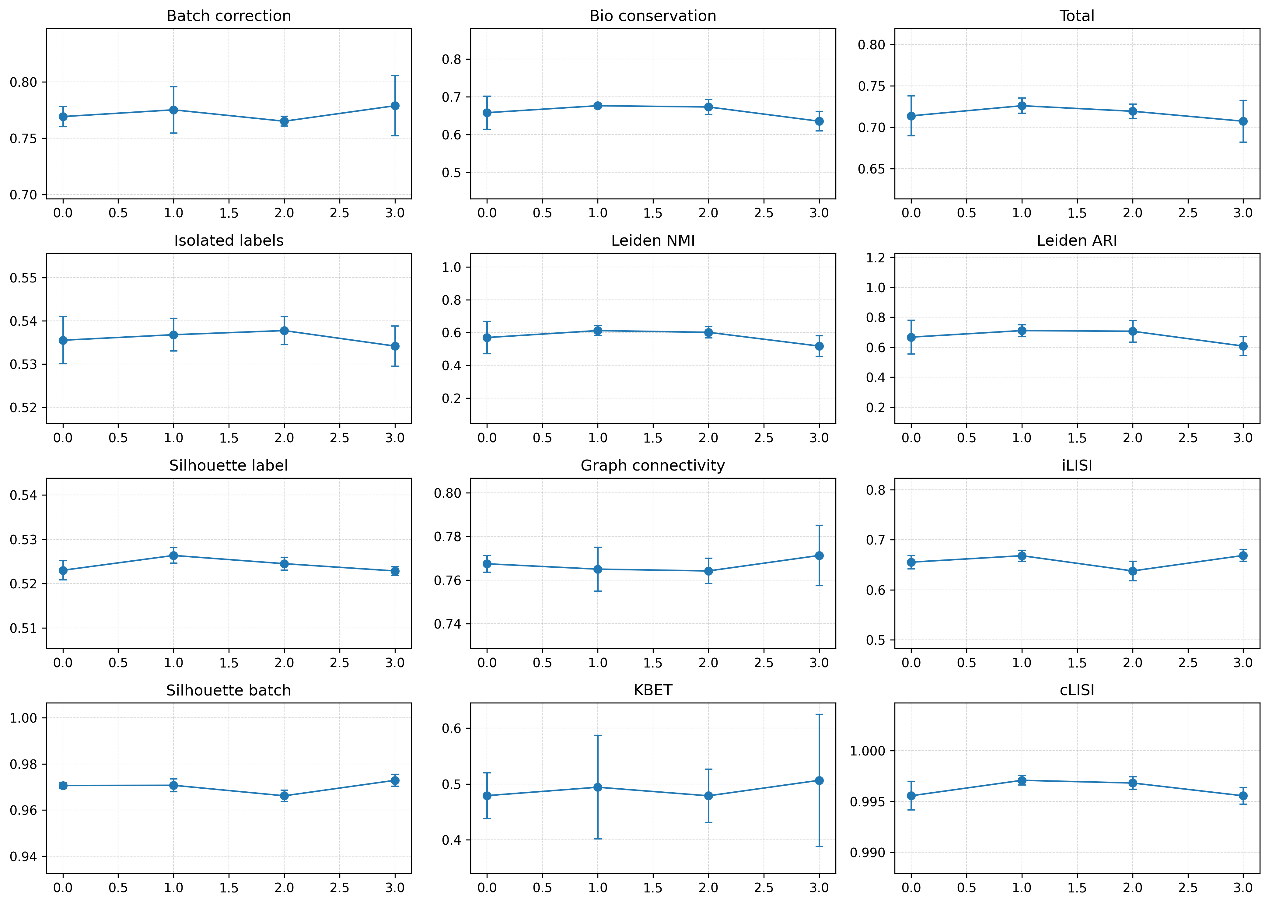


**Supplementary Figure S19 | Robustness of GAMMI to perturbations in prior feature–feature associations**

Each panel shows the mean (line) and standard deviation (error bars) of the corresponding evaluation metric across different levels of prior perturbation, where feature–feature association networks are progressively degraded by randomly removing or weakening prior edges. Performance is evaluated using standard batch correction, biological conservation, and clustering-related metrics. Across all perturbation levels, GAMMI exhibits consistently stable performance without systematic degradation, indicating that the model does not rely critically on the completeness or accuracy of prior knowledge and remains robust under partially incorrect or incomplete priors.

**Supplementary Table S6.** Comparison of representative single-cell and spatial multi-omics integration methods

| Method | Data Structure | Unpaired | Mosaic | Missing Modalities | Graph | Spatial |
| --- | --- | --- | --- | --- | --- | --- |
| Seurat | scRNA + multi-modal single-cell | ⚠️ | ⚠️ | ⚠️ | ❌ | ❌ |
| Tangram | scRNA + spatial | ❌ | ❌ | ❌ | ❌ | ✅ |
| stLearn | Spatial transcriptomics | ❌ | ❌ | ❌ | ✅ | ✅ |
| cell2location | scRNA + spatial | ❌ | ❌ | ❌ | ❌ | ✅ |
| GLUE | scRNA + scATAC | ✅ | ⚠️ | ⚠️ | ✅ | ❌ |
| MOFA+ | Multi-omics | ✅ | ⚠️ | ✅ | ❌ | ❌ |
| LIGER | Multi-omics | ✅ | ⚠️ | ⚠️ | ❌ | ❌ |
| MultiVI | scRNA + scATAC | ⚠️ | ⚠️ | ✅ | ❌ | ❌ |
| Cobolt | Multi-omics | ✅ | ⚠️ | ⚠️ | ❌ | ❌ |
| scMoMaT | Mosaic multi-omics | ✅ | ✅ | ✅ | ❌ | ❌ |
| StabMap | Multi-omics | ✅ | ⚠️ | ⚠️ | ❌ | ❌ |
| **GAMMI (ours)** | **Mosaic multi-omics + spatial** | ✅ | ✅ | ✅ | ✅ | ✅ |

⚠️ indicates partial support, restricted support, or support only under specific settings rather than as the primary intended design of the method.
